# Differences in the Gut Microbiota Establishment and Metabolome Characteristics Between Low- and Normal-Birth-Weight Piglets During Early-Life

**DOI:** 10.3389/fmicb.2018.01798

**Published:** 2018-09-07

**Authors:** Na Li, Shimeng Huang, Lili Jiang, Wei Wang, Tiantian Li, Bin Zuo, Zhen Li, Junjun Wang

**Affiliations:** ^1^State Key Laboratory of Animal Nutrition, College of Animal Science and Technology, China Agricultural University, Beijing, China; ^2^Beijing Advanced Innovation Center for Food Nutrition and Human Health, China Agricultural University, Beijing, China; ^3^State Key Laboratory of Plant Physiology and Biochemistry, College of Biological Sciences, China Agricultural University, Beijing, China

**Keywords:** low-birth-weight, fecal metabolites, microbiota, metabolite, piglet

## Abstract

Low-birth-weight (LBW) piglets are at a high-risk for postnatal growth failure, mortality, and metabolic disorders later in life. Early-life microbial exposure is a potentially effective intervention strategy for modulating the health and metabolism of the host. Yet, it has not been well elucidated whether the gut microbiota development in LBW piglets is different from their normal littermates and its possible association with metabolite profiles. In the current study, 16S rRNA gene sequencing and metabolomics was used to investigate differences in the fecal microbiota and metabolites between LBW and normal piglets during early-life, including day 3 (D3), 7 (D7), 14 (D14), 21 (D21, before weaning), and 35 (D35, after birth). Compared to their normal littermates, LBW piglets harbored low proportions of *Faecalibacterium* on D3, *Flavonifractor* on D7, *Lactobacillus*, *Streptococcus*, and *Prevotella* on D21, as well as *Howardella* on D21 and D35. However, the abundance of *Campylobacter* on D7 and D21, *Prevotella* on D14 and D35, *Oscillibacter* and *Moryella* on D14 and D21, and *Bacteroides* on D21 was significantly higher in LBW piglets when compared with normal piglets. The results of the metabolomics analysis suggested that LBW significantly affected fecal metabolites involved in fatty acid metabolism (e.g., linoleic acid, α-linolenic acid, and arachidonic acid), amino acid metabolism (e.g., valine, phenylalanine, and glutamic acid), as well as bile acid biosynthesis (e.g., glycocholic acid, 25-hydroxycholesterol, and chenodeoxycholic acid). Spearman correlation analysis revealed a significant negative association between *Campylobacter* and N1-acetylspermine on D7, *Moryella* and linoleic acid on D14, *Prevotella* and chenodeoxycholic acid on D21, and *Howardella* and phenylalanine on D35, respectively. Collectively, LBW piglets have a different gut bacterial community structure when compared with normal-birth-weight (NBW) piglets during early-life, especially from 7 to 21 days of age. Also, a distinctive metabolic status in LBW piglets might be partly associated with the altered intestinal microbiota. These findings may further elucidate the factors potentially associated with the impaired growth and development of LBW piglets and facilitate the development of nutritional interventions.

## Introduction

Genetic selection for high-prolific sows has substantially increased litter size over the last few decades (Yuan et al., 2015). However, larger litters are closely correlated with an increasing prevalence of LBW piglets (Quesnel et al., 2008) due to IUGR (Wu et al., 2006). Piglets with a birth weight of less than 1.1 kg are defined as LBW, which accounts for 15–25% of neonatal piglets (Li et al., 2017; Wang et al., 2017). LBW piglets are more prone to neonatal deaths, postnatal growth restriction, as well as poor carcass quality (Wu et al., 2006; Berard et al., 2008). In addition, long-term dysfunctions in vital organs are observed in LBW piglets, especially the impaired development of the GIT (Wang et al., 2008, 2014; Li et al., 2017).

The GIT in mammals harbors a large microbial community (Rooks and Garrett, 2016). The early-life development of the gut microbiota is believed to be paramount for the early-stage maturation of gut barrier function, the innate immune system, and the health of the host (Matamoros et al., 2013; Kabat et al., 2014). The gut microbiota in neonates is extremely turbulent, and it is shaped by many environmental factors, including host genetics (Goodrich et al., 2014), delivery mode (Wang et al., 2013), dietary change (Li et al., 2012; Bian et al., 2016), and feeding environment (Inman et al., 2010; Schokker et al., 2014). A dysbiosis in the gut microbial community of neonates not only results in a higher risk of diseases but also causes short- and long-lasting adverse effects on health (Foxx-Orenstein and Chey, 2012; Saavedra and Dattilo, 2012).

Many studies have reported the early-life development of the gut microbiota in newborn piglets born with NBW (Frese et al., 2015; Bian et al., 2016; Hu et al., 2016; Chen et al., 2017; Mu et al., 2017), while few studies have focused on the gut bacterial succession in LBW piglets during early-life. Previous studies have shown that counts of adherent bacteria via a traditional colony-counting method were greater in the intestinal mucosa of 2- to 5-day-old piglets with IUGR, when compared with the normal ones (D’Inca et al., 2010, 2011). However, these studies did not characterize the taxonomic composition of the bacterial community or assess its dynamic changes during the early-life of piglets. Therefore, differences in the gut microbiota of LBW and NBW piglets in the early stages after birth need to be further studied.

One of the mechanisms through which the gut microbiota indirectly impacts host physiology is the production of microbial metabolites and modulation of host immunity (Rooks and Garrett, 2016; Turroni et al., 2018). Metabolic disorders caused by the gut microbiota are often associated with disease such as insulin resistance, diabetes, and inflammation (Zmora et al., 2017; Turroni et al., 2018). For example, metabolomic alterations were observed in the small intestine of 21-day-old piglets suffering from IUGR (He et al., 2011). To our knowledge, information about the changes in intestinal microbiota-related metabolites in LBW piglets is not available.

We hypothesized that the gut microbiota development and metabolism in LBW piglets were different from their normal littermates during early-life. Therefore, the present study was designed to investigate differences in the gut microbiota composition and fecal metabolome between LBW and NBW piglets during early-life, including the preweaning and postweaning period. Possible associations between microbes and metabolites were also revealed. The results of this study will help to further understand the negative effects on growth performance in LBW piglets and facilitate the exploration of new therapeutic biomarkers for newborns with LBW.

## Materials and Methods

### Ethics Statement

All experimental protocols were carried out with the approval of the China Agricultural University Animal Care and Use Committee (CAU20170114-1, Beijing, China).

### Animal Management and Fecal Sampling

In this study, a total of 30 multiparous sows (Yorkshire; 2∼4 parities) were selected and raised individually in a commercial pig breeding farm in Sichuan province, China. During the entire experimental period, sows were fed the same commercial feed and water was provided *ad libitum* from nipple drinkers. Thirty litters of piglets (Landrace × Yorkshire) were spontaneously delivered from sows after 113∼114 days of gestation. At birth, 1 LBW piglet (0.75∼0.95 kg) and 1 NBW piglet (1.35∼1.55 kg) were obtained from each of the thirty litters (each litter with 7∼11 piglets). No cross-fostering was used in this study. From day 3 to 5 after birth, piglets started to receive commercial creep feed and drinking water *ad libitum*. All the piglets were weaned at 21 days of age and transferred into the nursery pens with free access to commercial weaning diet and water. None of the piglets were administered with antibiotics or other drugs throughout this experiment.

Body weights of all newborn piglets were recorded immediately after delivery. On day 3 (D3), 7 (D7), 14 (D14), 21 (D21, before weaning), and 35 (D35, after birth), piglets (6 LBW and 6 NBW piglets) from each of the six litters were weighed individually after 2∼4 h of fasting and then sacrificed after anesthesia for sample collection. To avoid contamination, fresh feces were collected from the terminal rectum of each piglet. A total of 60 fecal samples from the rectum were collected on ice, immediately frozen in liquid nitrogen, and then stored at -80°C until microbiome and metabolome analysis.

### DNA Extraction, 16S rRNA Gene Amplification and Sequencing

Total metagenomic DNA was extracted from 200 mg of each fecal specimen by using the QIAamp^®^ Fast DNA Stool Mini Kit (Qiagen Ltd., Germany) in accordance with manufacturer’s instructions. The V3-V4 region of the 16S rRNA gene was amplified with universal primers 341F (ACTCCTACGGGAGGCAGCAG) and 806R (GGACTACHVGGGTWTCTAAT), as described by Hong et al. (2016). The amplified products were detected using agarose gel electrophoresis (2% agarose), recovered by AxyPrep DNA Gel Recovery Kit (Axygen Biosciences, Union City, CA, United States), and then quantified by Qubit 2.0 Fluorometer (Thermo Fisher Scientific, Waltham, MA, United States) to pool into equimolar amounts. Amplicon libraries were sequenced on the Illumina HiSeq 2500 platform (Illumina, San Diego, CA, United States) for paired-end reads of 250 bp. All the raw data involved in the present study were deposited in NCBI Sequence Read Archive (SRA) under accession number SRP137635.

### Analysis of Sequencing Data

The raw paired-end reads were assembled into longer sequences and quality filtered by PANDAseq (version 2.9) to remove the low-quality reads with a length of <220 nucleotides (nt) or >500 nt, an average quality score of <20, and sequences containing >3 nitrogenous bases (Masella et al., 2012). The high-quality sequences were clustered into OTUs with a 97% similarity using UPARSE (version 7.0) (Edgar, 2013) in QIIME (version 1.8) (Caporaso et al., 2010), and the chimeric sequences were removed using UCHIME (Edgar et al., 2011). Taxonomy was assigned to OTUs using the RDP classifier^1^ (Bacci et al., 2015) against the SILVA 16S rRNA gene database (Release128^2^) (Pruesse et al., 2007), with a confidence threshold of 70%.

The Shannon diversity index and the number of OTUs per sample were calculated by the MOTHUR program (version v.1.30.1^3^) (Schloss et al., 2009). Bar plots and heat maps were generated with the “vegan” package in R (version 3.3.1). For beta diversity analysis, PCoA was performed based on Bray–Curtis distances using QIIME (version 1.8).

### Fecal Metabolite Extraction and UPLC-MS Analysis

A total of 60 fecal samples were analyzed in the UPLC-MS platform according to the protocol described in a previous study (Cao et al., 2016). In brief, each fecal sample (∼100 mg) was mixed with 400 μL MeOH:ACN (1:1, v/v, 4°C), followed by centrifugation at 15, 000 × *g* for 10 min. The supernatant was transferred to another 1.5 mL centrifuge tube and dried in a vacuum concentrator (Concentrator plus, Eppendorf). Next, the dried extracts were separately redissolved with 200 μL MeOH:H_2_O (4:1, v/v, 4°C), and then centrifuged at 15, 000 × *g* for 10 min. The final supernatant was filtered through a 0.22 μm sterile membrane and proceeded using a UPLC-HRMS system (UPLC, ACQUITYUPLC H-Class Bio, Waters; MS, Q-Exactive, Thermo Scientific), equipped with a HESI source under the standard procedures.

### Metabolomics Data Processing

Raw data processing and further data analysis were conducted according to a previous publication (Cao et al., 2016). In brief, SIEVE 2.1 software (Thermo Fisher Scientific, NJ, United States) was applied for peak alignment, background exclusion, and component extraction of raw data. Component extraction was achieved between retention times 0.5 and 16 min, with intensity threshold at 500,000, minimum scan at 9, and signal-to-noise ratio of 10. OPLS-DA was generated using SIMCA-P 13 software (Umetrics, Umea, Sweden) after data were scaled to Pareto variance. Compounds with a criterion of CV <20%, fold change >1.5, and *P* < 0.05 were filtered as differential metabolites between two groups, using EXCEL for further identification. The identification of these candidate metabolites was carried out using the Human Metabolome Database^4^ and METLIN^5^ based on the exact masses of molecular ions. The MS/MS spectra database was used to match fragment ion spectra of the candidate compounds. Similarly, MS/MS spectra were also compared with theoretical fragmentation patterns with mass tolerance at 5 ppm using Xcalibur^TM^ (Thermo Fisher Scientific, NJ, United States). The impacts of birth weight on metabolic pathways and metabolite set enrichment analysis (MSEA) were analyzed using MetaboAnalyst 4.0^6^ (Chong et al., 2018).

### Statistical Analysis

The difference in growth performance between LBW and NBW piglets at different time-points was tested using the GLM (SPSS 20.0). Both age and birth weight were considered as fixed factors, and means were separated and adjusted using Duncan’s multiple test. *P*-values below 0.05 were considered statistically significant. The difference in the alpha diversity between LBW and NBW piglets at each time-point was tested using Mann–Whitney *U*-test (SPSS 20.0), and *P*-values were adjusted with FDR (below 5%) as described by Benjamini and Hochberg (1995). The corrected *P*-values below 0.05 were regarded as statistically significant. To compare the difference in the gut microbiota structure between LBW and NBW piglets at different time-points, PERMANOVA (1, 000 Monte Carlo permutations) was performed based on Bray–Curtis distances with the Adonis function available in the package “vegan” in R (version 3.3.1) (Anderson and Walsh, 2013). Linear discriminant analysis (LDA) effect size (LEfSe) analysis was used to identify the differential genera between LBW and NBW groups. Only genera with an average relative abundance greater than 0.01% were considered. Correlations between different metabolites and bacterial communities were assessed by Spearman’s correlation analysis using the “pheatmap” package in R (version 3.3.1). Data were expressed as mean values.

## Results

### Effects of Birth Weight on Growth Performance in Piglets

Low-birth-weight piglets continuously showed a significantly lower body weight (*P* < 0.001) than NBW piglets during the whole experimental period (**Supplementary Table S1**). At D35, the body weight of LBW piglets was 17% lower than that of the normal ones. Furthermore, from D3 to D21, LBW piglets had a lower ADG when compared with NBW piglets (*P* < 0.001).

### Summary of 16S rRNA Gene Profiles and Alpha Diversities Across All the Samples

A total of 3, 136, 875 high-quality 16S rRNA gene sequences were generated from sixty fecal samples. We randomly subsampled all the samples to 30, 996 sequences to avoid bias caused by different sequencing depth. Based on 97% sequence similarity, 1, 076 OTUs were identified and then assigned to 17 phyla, 32 classes, 54 orders, 89 families, and 264 genera.

**FIGURE 1:**
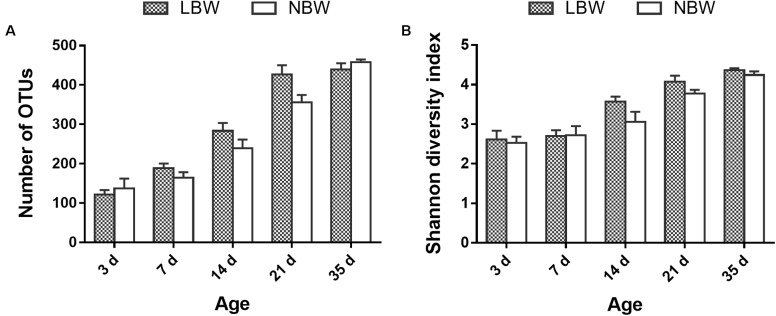
Alpha diversity of the gut bacterial community of LBW and NBW piglets at each sampling time. **(A)** Number of OTUs and **(B)** Shannon diversity index of LBW and NBW piglets at each time-point. Data are shown as mean ± SEM. *n* = 6 per group. LBW, low-birth-weight; NBW, normal-birth-weight.

The difference in gut bacterial diversity and richness between LBW and NBW piglets is shown in **Figure 1**. Birth weight had no significant influence on fecal bacterial richness and diversity (number of OTUs and Shannon diversity index) at any tested time-point (**Figures 1A,B**).

### Age-Induced Changes in the Gut Microbiota of Piglets From Preweaning to Postweaning Period

**FIGURE 2:**
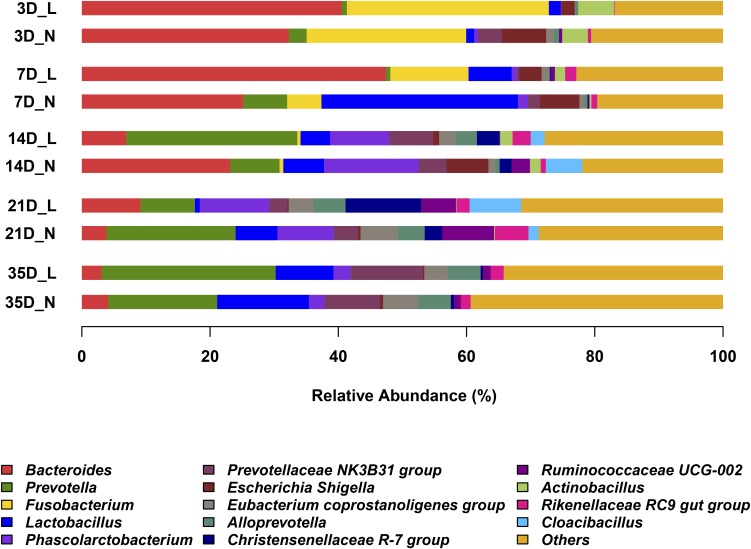
Abundant genera in the gut microbiota of LBW and NBW piglets. Only genera with average relative abundance greater than 5% were shown. Data are shown as means in each group, *n* = 6 per group. L, low-birth-weight; N, normal-birth-weight.

**FIGURE 3:**
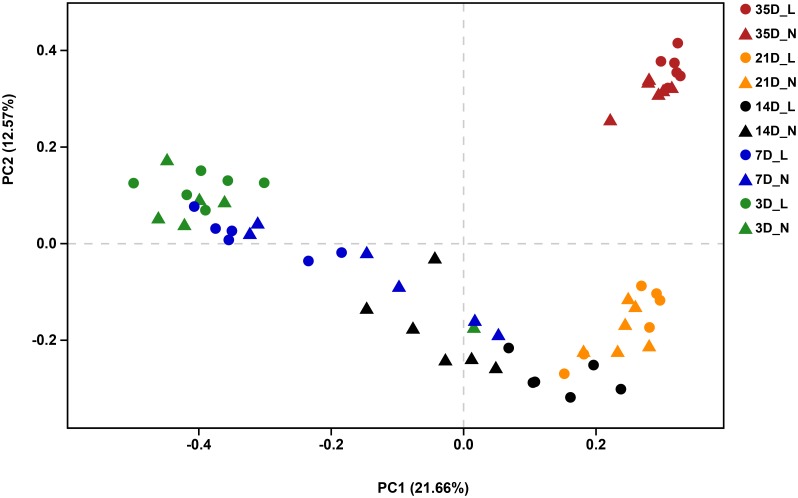
Principal coordinates analysis (PCoA, Bray–Curtis distance) plot of the gut microbial community structure by sampling time and birth weight status. *n* = 6 per group. L, low-birth-weight; N, normal-birth-weight.

The overall bacterial composition of the piglet gut varied significantly by age, irrespective of birth weight (**Figure 2** and **Supplementary Figure S1**). A PCoA plot of the Bray–Curtis distances confirmed that samples clustered primarily in an age-dependent manner along the PC1 axis (**Figure 3**). A PERMANOVA of these distances also showed that the gut microbiota structure was strongly affected by age (*R*^2^ = 0.382, *P* = 0.001). At the phylum level (**Supplementary Figure S1**), Bacteroidetes was predominant at all time-points, with the mean relative abundance ranging from 42.0–51.9%. The relative abundance of Firmicutes was lower on D3 (16.8%) when compared to D7 (36.1%). Fusobacteria and Proteobacteria were the dominant phyla on D3 but the levels decreased on D7. Furthermore, Fusobacteria was nearly undetectable on D21 and D35. At the genus level (**Figure 2**), *Bacteroides*, *Fusobacterium*, and *Escherichia-Shigella* were the main bacterial genera in the fecal samples from D3 to D7. In contrast, *Prevotella*, *Phascolarctobacterium*, *Prevotellaceae NK3B3E* group and *Alloprevotella* were dominant in the gut from D14 to D35. *Lactobacillus* had a lower relative abundance on D3 (1.6%) but was predominant at other ages, with the highest value on D7 (18.7%). The relative abundances of the top 50 most abundant genera are shown in **Supplementary Figure S2**.

### Differences in the Gut Microbiota Between LBW and NBW Piglets During Early-Life

We visualized the Bray–Curtis distances using PCoA and statistically with PERMANOVA to evaluate the effect of birth weight status on the bacterial community structure. The PCoA plot revealed distinct separation of the bacterial community structure by birth weight on D7, D14, and D21 (**Supplementary Figures S3B,C,D**), which was confirmed by PERMANOVA (D7, *R*^2^ = 0.182, *P* = 0.009; D14, *R*^2^ = 0.169, *P* = 0.012; D21, *R*^2^ = 0.157, *P* = 0.017). However, the microbial community structure was relatively similar between LBW and NBW piglets on D3 (**Supplementary Figure S3A**; *R*^2^ = 0.145, *P* = 0.084) or D35 (**Supplementary Figure S3E**; *R*^2^ = 0.116, *P* = 0.077).

**FIGURE 4:**
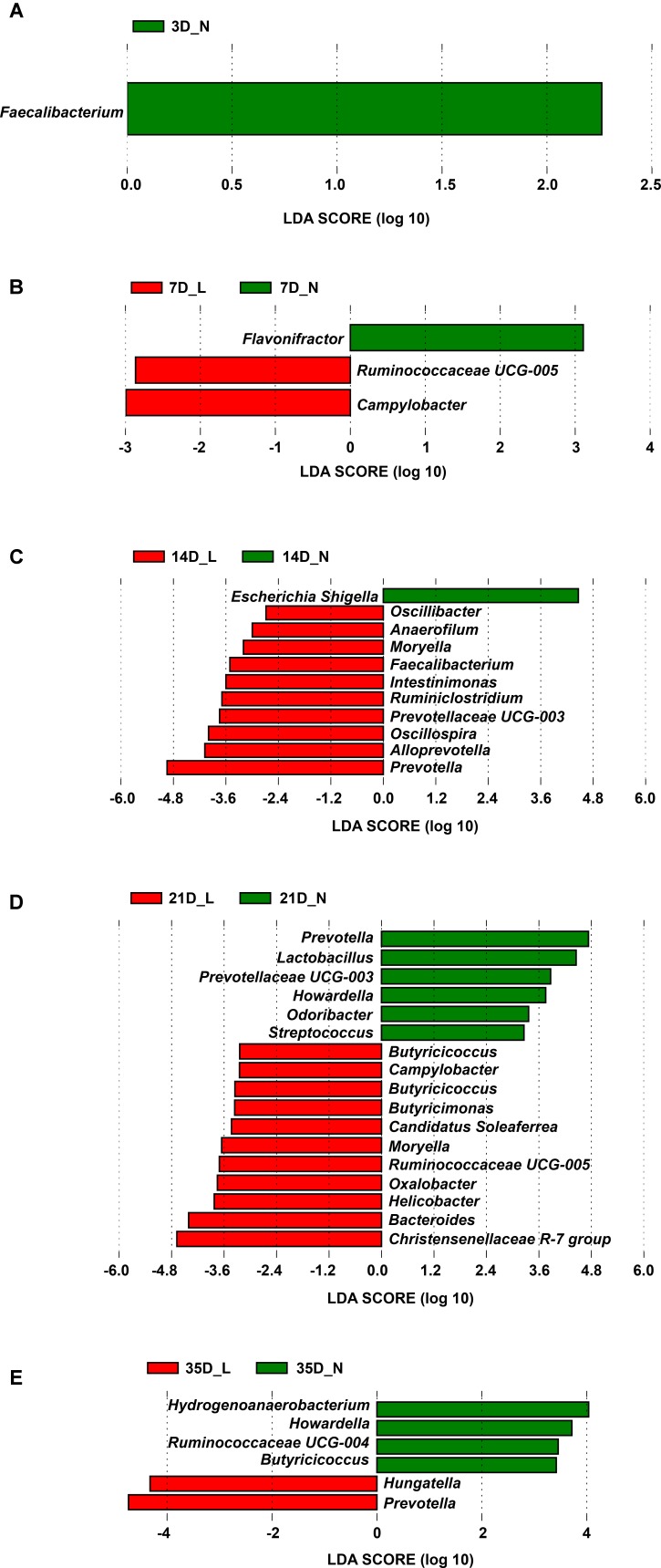
Differentially abundant genera between LBW and NBW piglets by sampling time. Histograms of a linear discriminant analysis (LDA) score (threshold ≥2) on D3 **(A)**, D7 **(B)**, D14 **(C)**, D21 **(D)**, and D35 **(E)** are plotted. *n* = 6 per group. L, low-birth-weight; N, normal-birth-weight.

Significant differences in the relative abundance of phyla and genera in the fecal microbiota between LBW and NBW piglets at a certain age were further identified using the Mann–Whitney *U*-test and LEfSe analysis (**Figure 4**, **Supplementary Figure S4**, and **Supplementary Table S2**). As the most dominant phylum, the relative abundance of the Bacteroidetes between LBW and NBW piglets was not significantly different at any time-point (*P* > 0.05, **Supplementary Figure S4B**). Compared with the NBW group, the relative abundance of the phylum Firmicutes was significantly lower (*P* < 0.05, **Supplementary Figure S4A**) in LBW piglets on D3 (mean, 11.9% vs. 21.7%) and D7 (mean, 25.9% vs. 46.2%). In contrast, the relative abundance of the phylum Fusobacteria (and the genus *Fusobacterium*) in LBW piglets was relatively high on D3 and D7, but the difference was not statistically significant (*P* > 0.05, **Supplementary Figures S4C,G**).

Results of LEfSe at the genus level revealed that one taxon on D3 was significantly impacted by birth weight, followed by three taxa on D7, eleven taxa on D14, seventeen taxa on D21, and six taxa on D35 (**Figure 4** and **Supplementary Table S2**). For example, on D3, LBW piglets showed a dramatically lower relative abundance of the genus *Faecalibacterium* (0.001% vs. 0.018%, *P* < 0.05) than NBW piglets. On D7, the relative abundances of the genus *Campylobacter* (0.074% vs. 0.001%, *P* < 0.05) and *Ruminococcaceae UCG-005* (0.059% vs. 0.003%, *P* < 0.05) were higher in LBW piglets, while a lower relative abundance of *Flavonifractor* (0.063% vs. 0.314%, *P* < 0.05) was observed in NBW piglets. A lower proportion of the genus *Escherichia-Shigella* (0.956% vs. 6.577%, *P* < 0.05) was observed in LBW piglets on D14 but a higher abundance of *Moryella* on D14 (0.020% vs. 0.000%, *P* < 0.05) and D21 (0.059% vs. 0.014%, *P* < 0.05) was seen in NBW piglets. On D21, an increased relative abundance of the genus *Bacteroides* (9.160% vs. 3.923%, *P* < 0.05) was observed for LBW piglets. Meanwhile, the relative abundance of *Campylobacter* (0.326% vs. 0.048%, *P* < 0.05) increased again as that on D7. Additionally, LBW piglets had a lower proportion of the genus *Howardella*, both on D21 (0.008% vs. 0.051%, *P* < 0.05) and D35 (0.012% vs. 0.055%, *P* < 0.05), when compared with NBW piglets. As the predominant genus, the proportion of the genus *Lactobacillus* was continuously lower in LBW piglets than in NBW piglets from D7 to D35, with a significant decrease on D21 (0.790% vs. 6.316%, *P* < 0.05, **Supplementary Figure S4D**). Compared to NBW piglets, the relative abundance of the genus *Prevotella* was significantly higher on D14 (26.343% vs. 7.276%, *P* < 0.05) and D35 (26.948% vs. 16.736%, *P* < 0.05) but significantly lower on D21 (8.342% vs. 19.499%, *P* < 0.05) in LBW piglets (**Supplementary Figure S4F**). The proportion of the genus *Streptococcus* was low in the LBW group during the suckling period (**Supplementary Figure S4H**), with a significant decline on D21 (0.087% vs. 0.243%, *P* < 0.05).

### Differences in Fecal Metabolite Profiles Between LBW and NBW Piglets During Early-Life

**FIGURE 5:**
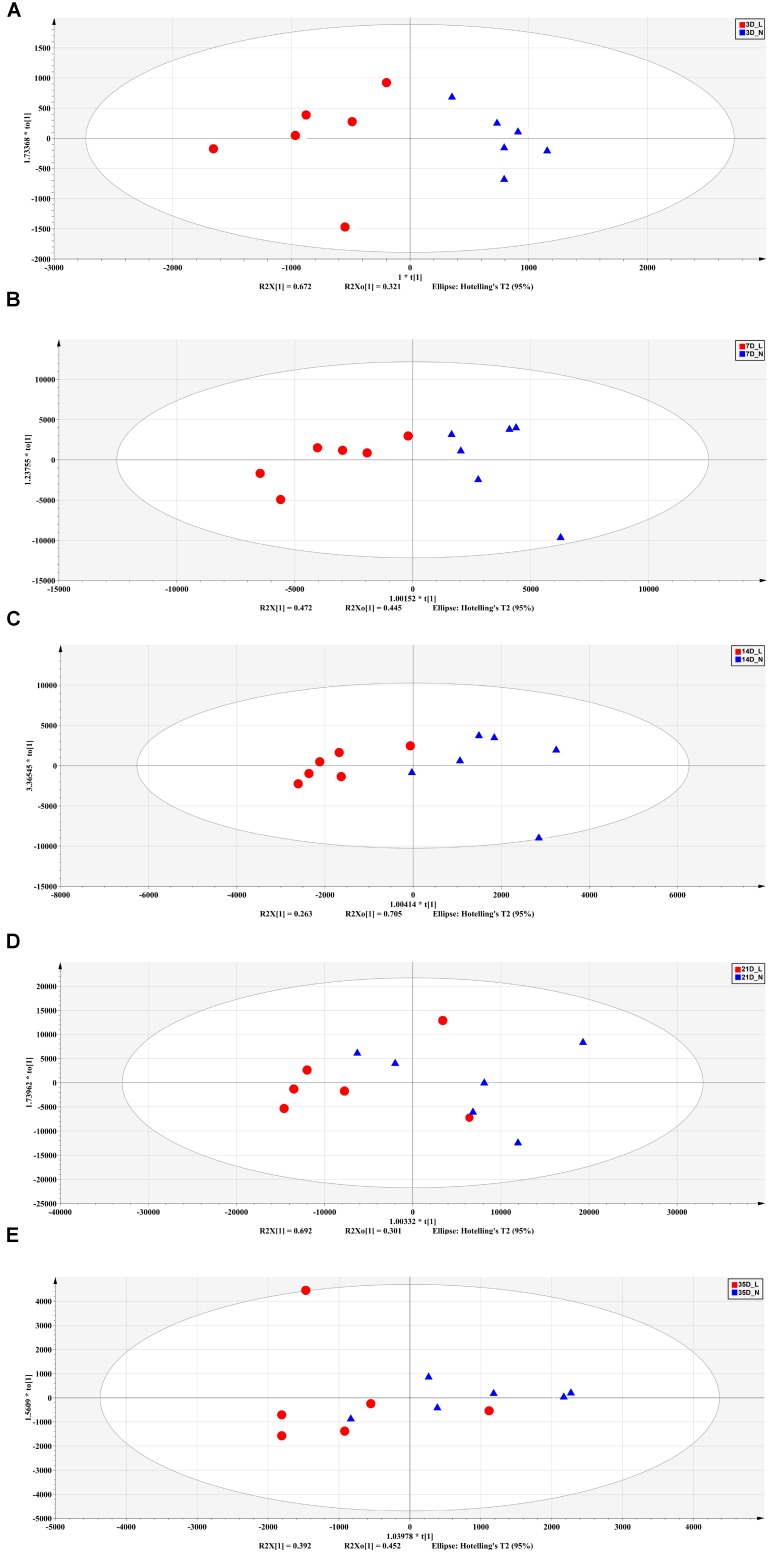
Orthogonal partial least squares discriminant analysis (OPLS-DA) plot of fecal metabolites between LBW and NBW piglets on D3 **(A)**, D7 **(B)**, D14 **(C)**, D21 **(D)**, and D35 **(E)**. *n* = 6 per group. L, low-birth-weight; N, normal-birth-weight.

**FIGURE 6:**
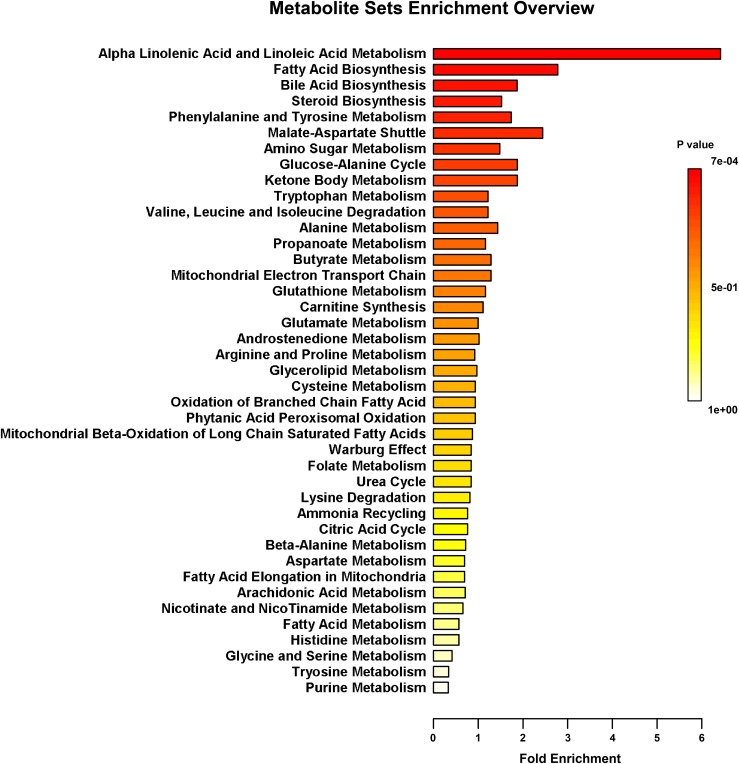
Metabolic pathway enrichment analysis. Overview of metabolites that were enriched in LBW piglets compared to the NBW ones. *n* = 6 per group.

The fecal metabolic profiles were analyzed by UPLC-MS. Differences in the metabolite profiles of LBW and NBW piglets at each time-point were revealed by OPLS-DA (**Figure 5**). A total of 46 differentially abundant metabolites were identified and annotated across the whole experimental period. These metabolites, which include amino acids, organic acids, fatty acids, and lipids, are involved in multiple biological pathways (**Supplementary Table S3**). Compared with the NBW group, LBW piglets had higher concentrations of glycocholic acid, L-valine, and vanilpyruvic acid on D3. On D7, nine compounds (linoleic acid, α-linolenic acid, palmitic acid, indoleacetic acid, α-dimorphecolic acid, cyclohexane undecanoic acid, 5,8-tetradecadienoic acid, 3-oxododecanoic acid, and cyclohexanecarboxylic acid) were significantly enriched, whereas the amounts of three metabolites (N1-acetylspermine, N-undecanoylglycine, and N-acetylcadaverine) were significantly declined in LBW piglets. It was noted that the concentrations of indoleacetic acid and α-dimorphecolic acid in the LBW group had a 7- and 6-fold increase, respectively. On D14, the production of ten metabolites (oleic acid, linoleic acid, kynurenic acid, indoleacetic acid, 2-phenylacetamide, deoxycholic acid, tetracosahexaenoic acid, myristoleic acid, 3-oxotetradecanoic acid, and cyclohexanecarboxylic acid) was downregulated in LBW piglets when compared with their NBW littermates. On D21, increased amounts of seven metabolites (25-hydroxycholesterol, chenodeoxycholic acid, arachidonic acid, stearoylcarnitine, zymosterol intermediate 2, docosahexaenoic acid, and desaminotyrosine) were observed in the LBW group, while three metabolites (desmosterol, phenylalanylphenylalanine, and 3-oxohexadecanoic acid) were reduced. In addition, on D35, LBW piglets had higher concentrations of etiocholanedione, 3-hydroxyhippuric acid, and L-phenylalanine. In contrast, lower values of eight metabolites (palmitic acid, L-glutamic acid, succinic acid, 3β,7α-dihydroxy-5-cholestenoate, allolithocholic acid, N-acetylneuraminic acid, hypogeic acid, and N-acetylserine) were found in LBW piglets. Further metabolite enrichment analysis indicated that LBW had a significant impact on fatty acid metabolism and biosynthesis, bile acid biosynthesis, and amino acid metabolism in piglets (**Figure 6**).

### Correlations Between the Fecal Microbial Composition and Metabolite Profiles

**FIGURE 7:**
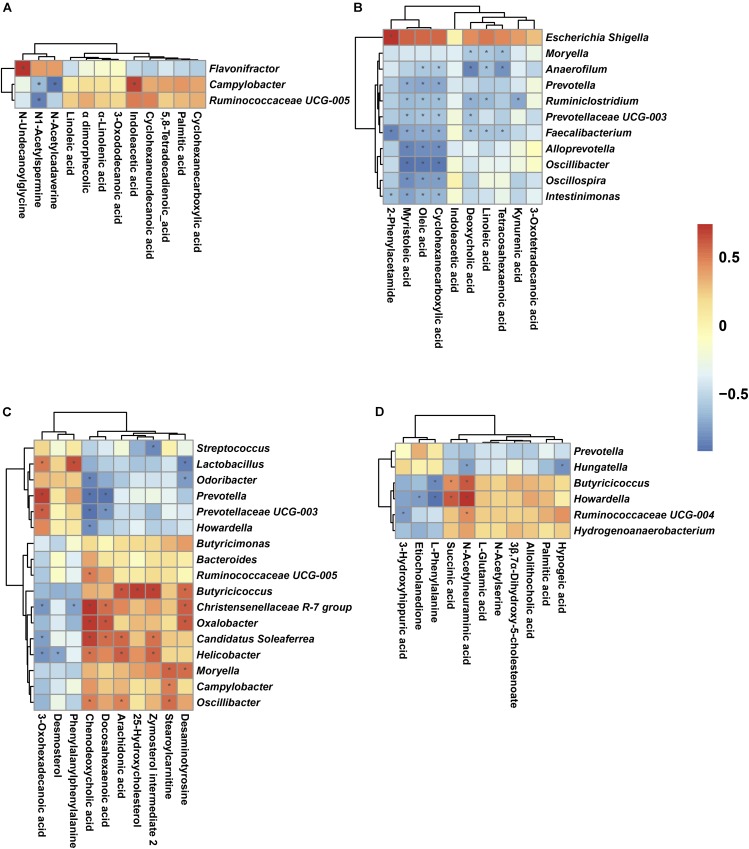
Spearman correlation analysis between genera and metabolite concentrations affected by birth weight on D7 **(A)**, D14 **(B)**, D21 **(C)**, and D35 **(D)**. Asterisks indicate significant correlations between LBW and NBW piglets. Cells are colored based upon the Spearman correlation coefficient between the significantly altered genera and metabolites; the red represents a significantly positive correlation (*P* < 0.05), the blue represents a significantly negative correlation (*P* < 0.05), and the white represents no significant correlation (*P* > 0.05). *n* = 6 per group.

A Spearman’s correlation matrix was generated to explore the correlation between the bacterial genera and candidate compounds that were significantly affected by birth weight. As shown in **Figure 7**, significant associations could be identified between the gut microbiota and the altered metabolite profiles from D7 to D35. On D7 (**Figure 7A**), the correlation analysis revealed that the genus *Flavonifractor* was positively correlated with N-undecanoylglycine (*R* = 0.79, *P <* 0.05). The genus *Ruminococcaceae UCG-005* was negatively associated with N1-Acetylspermine (*R* = -0.83, *P* < 0.05). The genus *Campylobacter*, having an increased abundance in LBW piglets, was positively correlated with indoleacetic acid (*R* = 0.73, *P <* 0.05) and negatively associated with N1-acetylspermine (*R* = -0.62, *P* < 0.05) and N-acetylcadaverine (*R* = -0.90, *P* < 0.05). On D14 (**Figure 7B**), 2-phenylacetamide was positively correlated with the genus *Escherichia-Shigella* (*R* = 0.74, *P* < 0.05) and negatively associated with the genera *Intestinimonas* (*R* = -0.63, *P* < 0.05) and *Faecalibacterium* (*R* = -0.85, *P* < 0.05). Oleic acid and cyclohexanecarboxylic acid were negatively associated with the same nine genera (these nine genera included *Anaerofilum*, *Prevotella*, *Ruminiclostridium*, *Prevotellaceae UCG-003*, *Faecalibacterium*, *Alloprevotella*, *Oscillibacter*, *Oscillospira*, *Intestinimonas*) (*R <* -0.58, *P* < 0.05) and myristoleic acid was negatively associated with eight genera (*R <* -0.62, *P* < 0.05). Linoleic acid had negative associations with four genera (*R <* -0.57, *P* < 0.05), deoxycholic acid with five genera (*R <* -0.57, *P* < 0.05), kynurenic acid with the genus *Ruminiclostridium* (*R* = -0.71, *P* < 0.05), and tetracosahexaenoic acid with three genera (*R <* -0.59, *P* < 0.05). On D21 (**Figure 7C**), ten genera (6 positive and 4 negative) were significantly correlated with chenodeoxycholic acid (*P* < 0.05); six genera (3 positive and 3 negative) were associated with 3-oxohexadecanoic acid (*P* < 0.05); five genera (3 positive and 2 negative) were associated with docosahexaenoic acid (*P* < 0.05); and three and four genera were positively associated with stearoylcarnitine and arachidonic acid, respectively (*R >* 0.58, *P* < 0.05). Similarly, desaminotyrosine, zymosterol intermediate 2, desmosterol, and phenylalanylphenylalanine were significantly correlated with six genera (4 positive and 2 negative), four genera (3 positive and 1 negative), one genera (negative), and two genera (1 positive and 1 negative), respectively (*P* < 0.05). For 35-day-old piglets (**Figure 7D**), the genus *Ruminococcaceae UCG-004* showed a negative correlation with 3-hydroxyhippuric acid (*R* = -0.59, *P* < 0.05) but a positive correlation with N-acetylneuraminic acid (*R* = 0.60, *P* < 0.05). The genus *Hungatella* presented negative associations with hypogeic acid and N-acetylneuraminic acid (*R* < -0.57, *P* < 0.05). The genera *Howardella* and *Butyricicoccus* showed negative associations with L-phenylalanine (*R* < -0.67, *P* < 0.05), whereas positive connections with succinic acid and N-acetylneuraminic acid (*R* > 0.59, *P* < 0.05).

## Discussion

Low-birth-weight piglets are strongly associated with high rates of postnatal mortality, reduced growth rates, and poor carcass quality (Wu et al., 2006; Berends et al., 2013). In the current study, we confirmed that LBW piglets had continuously poor growth performance during early-life, which was consistent with previous findings (Wang et al., 2010; Douglas et al., 2014; Hu et al., 2015). In addition, we systematically identified differentially abundant bacterial genera and metabolites between LBW and NBW piglets, from birth through the postweaning period, using a microbiota-metabolome analysis. Our findings showed that the dynamic establishment of the gut microbiota was strongly affected by age, and LBW was associated with alterations in the gut microbial community and fecal metabolome of piglets during the suckling and weaning period. Furthermore, associations existed between the differentially abundant genera and metabolites in the LBW and NBW groups.

### Establishment of the Gut Microbial Community in Newborn Piglets

In the present study, Firmicutes and Bacteroidetes were the two most predominant phyla in piglet gut microbiota, which was consistent with previous studies in piglets (Bian et al., 2016; Hu et al., 2016; Chen et al., 2017) and human infants (Backhed et al., 2015; Kostic et al., 2015). Fusobacteria was dominant on D3 but disappeared after weaning, which was also observed in other studies (Bian et al., 2016; Chen et al., 2017). Proteobacteria, which include a wide variety of pathogenic bacteria, also showed a significant decline with age, and this observation was in agreement with previous studies (Zhao et al., 2015; Bian et al., 2016; Hu et al., 2016; Chen et al., 2017). These results suggested that potential pathogens might be mainly present in the newborn GIT, and therefore make newborns more susceptible to disease. At the genus level, the relative abundance of *Fusobacterium* sharply reduced from the suckling to weaning period as reported in other studies (Chen et al., 2017). *Lactobacillus* was predominant in the gut microbiota of piglets from D7 to D35 in our study, which was in accordance with studies carried out by Bian et al. (2016) and Mach et al. (2015). Bacterial richness and diversity increased over time, and this finding was in accordance with other studies in piglets (Frese et al., 2015; Mach et al., 2015; Bian et al., 2016; Chen et al., 2017) and human infants (Matamoros et al., 2013; Backhed et al., 2015; Hill et al., 2017).

### Differences in the Gut Microbiota Between LBW and NBW Piglets During Early-Life

Evidence has shown that bacterial establishment in the gut can be altered in premature infants born with LBW during early-life (Fanca-Berthon et al., 2010; Arboleya et al., 2012a,b). Studies in piglets have reported greater counts of adherent bacteria in the intestinal mucosa of 2- to 5-day-old piglets with IUGR (D’Inca et al., 2010, 2011). In the current study, no clear separation was observed in the gut microbial community structure of LBW and NBW piglets on D3. Immediately after birth, neonates experience a transition from an intrauterine environment to an external environment. The fetus is in a relatively sterile aquatic environment during pregnancy and depends mainly on the umbilical vein for parenteral nutrition. Immediately after birth, the neonate begins enteral nutrition by suckling maternal milk and the GIT is colonized with microbes. Therefore, rapid changes in environment and nutrition during the early postnatal period may lead to high intraindividual variability in the gut microbiota of piglets, and these changes explain the lack of significant differences between the LBW and NBW piglets on D3. Our results revealed that the bacterial community structure of LBW and NBW piglets showed significant separation on D7, D14, and D21, but not on D35. Impaired development of the GIT (Wang et al., 2010) and insufficient milk intake (Quesnel et al., 2012) in LBW piglets, might be the two factors that explain the subsequent dissimilarity in the structure of the gut microbiota between the two groups. After weaning, the large changes in the environment and diet may result in a similar microbial community structure for both LBW and NBW piglets.

Furthermore, our findings demonstrated that birth weight affected specific bacterial taxa at all time-points, especially from D7 to D21. This suggests that the suckling period may be the critical developmental period for the gut microbiota and have long-lasting impacts on health and metabolism. A lower relative abundance of Firmicutes was harbored by LBW piglets on D3 and D7, which was in agreement with the fact that the relative abundance of Firmicutes decreased in the placenta from LBW infants (Zheng et al., 2015). Also, in the present study, LBW piglets had a lower abundance of *Lactobacillus* and *Streptococcus* but a higher abundance of *Fusobacterium*, which was consistent with previous studies in LBW infants (Arboleya et al., 2012a; Zheng et al., 2015) and rodents (Wang et al., 2016). LBW piglets are well recognized as being more susceptible to GIT defects and various diseases (Li et al., 2017; Wang et al., 2017). Some *Fusobacterium* spp. are pathogenic in pigs (De Witte et al., 2017), whereas *Lactobacillus* spp. are widely used as probiotics to improve health and disease resistance (Naito et al., 2011; Wang et al., 2012), and *Streptococcus* spp. are commensal members in the gut of newborns (Matamoros et al., 2013). Thus, the observed alterations in the members of these genera may partly explain the high morbidity of LBW piglets. Additionally, the relative abundances of *Faecalibacterium* on D3 and *Prevotella* on D21 were significantly decreased in the LBW group when compared with the NBW group. *Prevotella* spp. are important acetate-producing bacteria (Ivarsson et al., 2014) and *Faecalibacterium* include many key butyrate-producing species (Benus et al., 2010), and these SCFAs improve intestinal barrier function and reduce inflammation in the gut (Liu et al., 2018; Turroni et al., 2018). It has been reported that 50% of butyrate-producing bacteria are able to produce butyrate from acetate (Barcenilla et al., 2000). The decline of these SCFA-producers might reflect an attenuated capacity to metabolize dietary fiber in LBW piglets.

*Campylobacter* spp., some of which are associated with diarrhea in piglets, were more abundant in LBW piglets than in NBW piglets on D7 and D21 (Yang et al., 2017). Also, LBW piglets had higher levels of *Moryella* and *Oscillibacter* on D14 and D21 when compared with the NBW group in our study. A recent study reported that adult mice with obesity had a higher relative abundance of *Moryella* (Sung et al., 2017). Similarly, Duca et al. (2014) found that the abundance of *Oscillibacter* was significantly higher in obese-prone rats when compared with obese-resistant rats. Low-birth-weight neonates have a higher risk for developing adult obesity (Li et al., 2017); therefore, whether *Moryella* and *Oscillibacter* are associated with obesity in the long-term in LBW neonates needs further investigation. In our study, LBW piglets had a higher proportion of *Prevotella* on D14 and D35 but a lower proportion of *Escherichia-Shigella* on D14 when compared with NBW littermates. However, similar results were also observed in 10-day-old piglets suffering from diarrhea (Yang et al., 2017). *Escherichia* spp., which include many opportunistic pathogens, have been shown to be negatively correlated with *Prevotella* in healthy children (Kang et al., 2013), diarrheic children (Pop et al., 2014), and healthy piglets (Yang et al., 2017). However, both our observations and the studies carried out by Yang et al. (2017) showed that no significant correlation existed between these two genera in diarrheic piglets. The *Escherichia-Shigella* spp. detected in the present study may not be pathogenic as diarrhea was not observed in either the LBW or NBW piglets.

### Changes in Metabolism Between LBW and NBW Piglets During Early-Life

In addition to alterations in the fecal microbiota of LBW piglets when compared to their NBW littermates, LBW also disrupted the metabolic status of neonatal piglets. Our metabolome data revealed that the discriminating metabolites in differentiating LBW and NBW piglets were mainly found on D7, D14, D21, and D35. At these time-points, 12, 10, 10, and 11, differential compounds in feces were identified, respectively. Similar to the observations in the bacterial community, only minor differences in metabolites were found in 3-day-old piglets, of which only three metabolites were identified.

The altered concentrations of 14 fatty acids in the feces of LBW piglets suggest a potential dysfunction in fatty acid biosynthesis and metabolism. Unsaturated and saturated fatty acids are used by the host for synthesizing triglycerides which can promote lipogenesis (Miyazaki et al., 2001). There is evidence that the levels of some metabolites associated with lipogenesis changed in neonatal piglets (He et al., 2011), fetal pigs (Lin et al., 2012), and human infants (Leduc et al., 2011) suffering from IUGR. It has been reported that LBW neonates have a higher risk for developing adult metabolic and cardiovascular disorders due to abnormal fat storage and lipid metabolism (Wu et al., 2006; Li et al., 2017). Thus, the observed alterations in these fatty acids may be an important factor in the development of metabolic diseases later in life in LBW neonates.

The concentrations of several amino acids in the feces of LBW piglets, such as L-valine, L-glutamic acid, and L-phenylalanine, were significantly different from that of NBW piglets. Amino acids serve as essential precursors to protein biosynthesis (Wu, 2009), and the alterations in amino acid composition indicate that body growth and development might be suppressed in LBW neonates due to the abnormality in intestinal protein synthesis (Wang et al., 2010). Specifically, we observed decreased levels of L-glutamic acid in LBW piglets on D35. Reduced concentrations of glutamine or glutamate have been reported in the small intestine of LBW piglets (He et al., 2011), umbilical plasma from LBW infants (Favretto et al., 2012; Ivorra et al., 2012), and fetal pigs (Lin et al., 2012). Glutamine plays a critical role in maintaining multiple important functions, such as nutrient metabolism, immune response, and intestinal integrity, as well as the synthesis of other bioactive compounds (Wu et al., 2007; Wu, 2010). Therefore, glutamine or glutamate supplementation in LBW piglets may be an effective way to improve growth and maintain gut health (Vaughn et al., 2003; Wu et al., 2011; Yuan et al., 2015). Another interesting observation in the present study is that the feces of LBW piglets have higher levels of L-phenylalanine and L-valine. However, previous studies revealed decreased levels of these two amino acids in the jejunum or umbilical plasma of LBW neonates (He et al., 2011; Favretto et al., 2012; Lin et al., 2012). These contradictory findings might have resulted from the differences in the intestinal region being sampled. Valine and phenylalanine are two indispensable amino acids for piglets and must be supplied in the diet (Lei et al., 2012; Rezaei et al., 2013); therefore, their enrichment in feces might indicate a lower bioavailability in the intestine of LBW piglets.

Additionally, we report, for first time, that birth weight status significantly alters the concentrations of several metabolites associated with bile acid biosynthesis, such as glycocholic acid, deoxycholic acid, 25-hydroxycholesterol, chenodeoxycholic acid, 3β,7α-dihydroxy-5-cholestenoate, and allolithocholic acid. Bile acids are signaling molecules essential for the absorption and metabolism of dietary lipids and fat-soluble vitamins (Chiang, 2009). Bile acids also interact with the intestinal microbiota (Wang et al., 2002) by regulating FXR and G protein-coupled bile acid receptor 1 (GPBAR1) signaling, which in turn maintain intestinal homeostasis (Fiorucci and Distrutti, 2015). Primary bile acids are derived from cholesterol in the liver and are converted to secondary bile acids by the intestinal microbiota (Ridlon et al., 2006). In the present study, we found that LBW piglets had higher levels of primary bile acids (glycocholic acid, 25-hydroxycholesterol, and chenodeoxycholic acid) but lower levels of secondary bile acids (deoxycholic acid). These findings might suggest a decreased conversion rate of bile acids from the primary to the secondary form in the intestine of LBW piglets. Also, bile acids might act as an opportunistic mediator for interventions in obesity and diabetes (Fiorucci and Distrutti, 2015). Disruptions in bile acid metabolism of LBW piglets might have a long-term adverse effect on their physiological behavior and health during postnatal life.

Diet and host physiological status have direct influences on host metabolism (He et al., 2011; Maier et al., 2017). Moreover, there is increasing evidence that a part of these impacts is regulated by the intestinal microbiota as many gut microbes are capable of producing bioactive molecules that affect host metabolism (Fiorucci and Distrutti, 2015). In the current study, Spearman’s correlation analysis revealed an association between the abundance of specific bacterial genera and metabolites that were significantly influenced by birth weight. The characterization of metabolic alterations modulated by the intestinal microbiota has been used to understand the molecular mechanisms of host health and disease development in humans and animals (Arrieta et al., 2015; Shi et al., 2015; Sun et al., 2016; Turroni et al., 2018). Altogether, the disruption of gut microbial composition and metabolic homeostasis could be a major underlying factor that induces stunted growth and development of LBW piglets.

## Conclusion

In summary, the present study revealed differences in the gut microbiota and metabolic status between LBW and NBW piglets during the suckling and weaning period. Firstly, birth weight status strongly affected the gut bacterial composition of piglets from 7 to 21 days of age but had no obvious impact on D3 because of larger individual variations. This finding implies that the suckling period might be the critical period for modulating the gut microbiota in LBW piglets and interventions beginning from D7 might be more effective. Secondly, LBW piglets had altered concentrations of metabolites involved in multiple biochemical processes, including fatty acid metabolism, bile acid biosynthesis, and amino acid metabolism. Moreover, these metabolic changes in LBW piglets might be partly modulated by the intestinal microbiota as there were associations between the abundance of specific bacterial genera and metabolites affected by birth weight. Collectively, these findings provide new directions in identifying the key factors affecting the early development of LBW piglets and formulating the corresponding nutritional intervention.

## Author Contributions

JW and NL designed the experiments. JW, NL, SH, and WW conducted the experiments. NL, SH, and LJ carried out the experiments and collected the samples. NL, SH, and BZ performed the analysis of samples. NL, TL, and ZL analyzed the data. NL, WW, and JW wrote and revised the manuscript. All authors read and approved the final manuscript.

## Conflict of Interest Statement

The authors declare that the research was conducted in the absence of any commercial or financial relationships that could be construed as a potential conflict of interest.

## Footnotes

1 http://rdp.cme.msu.edu/

2 http://www.arb-silva.de

3 http://www.mothur.org

4 http://www.hmdb.ca

5 http://metlin.scripps.edu

6 http://www.metaboanalyst.ca

## Abbreviations

ADG: average daily gain
FDR: false discovery rate
FXR: farnesoid X receptor
GIT: gastrointestinal tract
GLM: general linear model
GPBAR1: G-protein-coupled bile acid receptor 1
HESI: heated electrospray ionization
IUGR: intrauterine growth restriction
LBW: low-birth-weight
LDA: linear discriminant analysis
LEfSe: linear discriminant analysis effect size
NBW: normal-birth-weight
OPLS-DA: orthogonal partial least squares discriminant analysis
OTUs: operational taxonomic units
PCoA: principal coordinates analysis
PERMANOVA: permutational multivariate analysis of variance
SCFAs: short-chain fatty acids
SEM: standard error of the mean
UPLC-MS: ultrahigh performance liquid chromatography-mass spectroscopy

## References

[B1] Anderson M. J. Walsh D. C. I. (2013). PERMANOVA, ANOSIM, and the mantel test in the face of heterogeneous dispersions: what null hypothesis are you testing? *Ecol. Monogr.* 83 557–574. 10.1890/12-2010.1

[B2] Arboleya S. Binetti A. Salazar N. Fernandez N. Solis G. Hernandez-Barranco A. (2012a). Establishment and development of intestinal microbiota in preterm neonates. *FEMS Microbiol. Ecol.* 79 763–772. 10.1111/j.1574-6941.2011.01261.x 22126419

[B3] Arboleya S. Solis G. Fernandez N. de los Reyes-Gavilan C. G. Gueimonde M. (2012b). Facultative to strict anaerobes ratio in the preterm infant microbiota: a target for intervention? *Gut Microbes* 3 583–588. 10.4161/gmic.21942 22922559 PMC3495798

[B4] Arrieta M. C. Stiemsma L. T. Dimitriu P. A. Thorson L. Russell S. Yurist-Doutsch S. (2015). Early infancy microbial and metabolic alterations affect risk of childhood asthma. *Sci. Transl. Med.* 7 307ra152. 10.1126/scitranslmed.aab2271 26424567

[B5] Bacci G. Bani A. Bazzicalupo M. Ceccherini M. T. Galardini M. Nannipieri P. (2015). Evaluation of the performances of Ribosomal Database Project (RDP) classifier for taxonomic assignment of 16s rrna metabarcoding sequences generated from illumina-solexa NGS. *J. Genom.* 3 36–39. 10.7150/jgen.9204 PMC4316179 25653722

[B6] Backhed F. Roswall J. Peng Y. Feng Q. Jia H. Kovatcheva-Datchary P. (2015). Dynamics and stabilization of the human gut microbiome during the first year of life. *Cell Host Microbe* 17 690–703. 10.1016/j.chom.2015.04.004 25974306

[B7] Barcenilla A. Pryde S. E. Martin J. C. Duncan S. H. Stewart C. S. Henderson C. (2000). Phylogenetic relationships of butyrate-producing bacteria from the human gut. *Appl. Environ. Microbiol.* 66 1654–1661. 10.1128/AEM.66.4.1654-1661.2000 10742256 PMC92037

[B8] Benjamini Y. Hochberg Y. (1995). Controlling the false discovery rate: a practical and powerful approach to multiple testing. *J. R. Stat. Soc. B.* 57 289–300.

[B9] Benus R. F. J. van der Werf T. S. Welling G. W. Judd P. A. Taylor M. A. Harmsen H. J. M. (2010). Association between *Faecalibacterium prausnitzii* and dietary fibre in colonic fermentation in healthy human subjects. *Br. J. Nutr.* 104 693–700. 10.1017/S0007114510001030 20346190

[B10] Berard J. Kreuzer M. Bee G. (2008). Effect of litter size and birth weight on growth, carcass and pork quality, and their relationship to postmortem proteolysis. *J. Anim. Sci.* 86 2357–2368. 10.2527/jas.2008-0893 18469061

[B11] Berends L. M. Fernandez-Twinn D. S. Martin-Gronert M. S. Cripps R. L. Ozanne S. E. (2013). Catch-up growth following intra-uterine growth-restriction programmes an insulin-resistant phenotype in adipose tissue. *Int. J. Obes.* 37 1051–1057. 10.1038/ijo.2012.196 PMC3734734 23229735

[B12] Bian G. Ma S. Zhu Z. Su Y. Zoetendal E. G. Mackie R. (2016). Age, introduction of solid feed and weaning are more important determinants of gut bacterial succession in piglets than breed and nursing mother as revealed by a reciprocal cross-fostering model. *Environ. Microbiol.* 18 1566–1577. 10.1111/1462-2920.13272 26940746

[B13] Cao J. Li M. Chen J. Liu P. Li Z. (2016). Effects of MeJA on *Arabidopsis* metabolome under endogenous JA deficiency. *Sci. Rep.* 6:37674 10.1038/srep37674 PMC5121592 27883040

[B14] Caporaso J. G. Kuczynski J. Stombaugh J. Bittinger K. Bushman F. D. Costello E. K. (2010). QIIME allows analysis of high-throughput community sequencing data. *Nat. Methods* 7 335–336. 10.1038/nmeth.f.303 20383131 PMC3156573

[B15] Chen L. Xu Y. Chen X. Fang C. Zhao L. Chen F. (2017). The maturing development of gut microbiota in commercial piglets during the weaning transition. *Front. Microbiol.* 8:1688 10.3389/fmicb.2017.01688 PMC5591375 28928724

[B16] Chiang J. Y. (2009). Bile acids: regulation of synthesis. *J. Lipid Res.* 50 1955–1966. 10.1194/jlr.R900010-JLR200 19346330 PMC2739756

[B17] Chong J. Soufan O. Li C. Caraus I. Li S. Bourque G. (2018). MetaboAnalyst 4.0: towards more transparent and integrative metabolomics analysis. *Nucleic Acids Res.* 46 W486–W494. 10.1093/nar/gky310 29762782 PMC6030889

[B18] De Witte C. Flahou B. Ducatelle R. Smet A. De Bruyne E. Cnockaert M. (2017). Detection, isolation and characterization of *Fusobacterium gastrosuis* sp. nov. colonizing the stomach of pigs. *Syst. Appl. Microbiol.* 40 42–50. 10.1016/j.syapm.2016.10.001 27816261

[B19] D’Inca R. Gras-Le Guen C. Che L. Sangild P. T. Le Huerou-Luron I. (2011). Intrauterine growth restriction delays feeding-induced gut adaptation in term newborn pigs. *Neonatology* 99 208–216. 10.1159/000314919 20881437

[B20] D’Inca R. Kloareg M. Gras-Le Guen C. Le Huerou-Luron I. (2010). Intrauterine growth restriction modifies the developmental pattern of intestinal structure, transcriptomic profile, and bacterial colonization in neonatal pigs. *J. Nutr.* 140 925–931. 10.3945/jn.109.116822 20335628

[B21] Douglas S. L. Edwards S. A. Kyriazakis I. (2014). Management strategies to improve the performance of low birth weight pigs to weaning and their long-term consequences. *J. Anim. Sci.* 92 2280–2288. 10.2527/jas.2013-7388 24671578

[B22] Duca F. A. Sakar Y. Lepage P. Devime F. Langelier B. Dore J. (2014). Replication of obesity and associated signaling pathways through transfer of microbiota from obese-prone rats. *Diabetes* 63 1624–1636. 10.2337/db13-1526 24430437

[B23] Edgar R. C. (2013). UPARSE: highly accurate OTU sequences from microbial amplicon reads. *Nat. Methods* 10 996–998. 10.1038/nmeth.2604 23955772

[B24] Edgar R. C. Haas B. J. Clemente J. C. Quince C. Knight R. (2011). UCHIME improves sensitivity and speed of chimera detection. *Bioinformatics* 27 2194–2200. 10.1093/bioinformatics/btr381 21700674 PMC3150044

[B25] Fanca-Berthon P. Hoebler C. Mouzet E. David A. Michel C. (2010). Intrauterine growth restriction not only modifies the cecocolonic microbiota in neonatal rats but also affects its activity in young adult rats. *J. Pediatr. Gastroenterol. Nutr.* 51 402–413. 10.1097/MPG.0b013e3181d75d52 20601908

[B26] Favretto D. Cosmi E. Ragazzi E. Visentin S. Tucci M. Fais P. (2012). Cord blood metabolomic profiling in intrauterine growth restriction. *Anal. Bioanal. Chem.* 402 1109–1121. 10.1007/s00216-011-5540-z 22101423

[B27] Fiorucci S. Distrutti E. (2015). Bile acid-activated receptors, intestinal microbiota, and the treatment of metabolic disorders. *Trends Mol. Med.* 21 702–714. 10.1016/j.molmed.2015.09.001 26481828

[B28] Foxx-Orenstein A. E. Chey W. D. (2012). Manipulation of the gut microbiota as a novel treatment strategy for gastrointestinal disorders. *Am. J. Gastroenterol. Suppl.* 1 41–46. 10.1038/ajgsup.2012.8

[B29] Frese S. A. Parker K. Calvert C. C. Mills D. A. (2015). Diet shapes the gut microbiome of pigs during nursing and weaning. *Microbiome* 3:28 10.1186/s40168-015-0091-8 PMC4499176 26167280

[B30] Goodrich J. K. Waters J. L. Poole A. C. Sutter J. L. Koren O. Blekhman R. (2014). Human genetics shape the gut microbiome. *Cell* 159 789–799. 10.1016/j.cell.2014.09.053 25417156 PMC4255478

[B31] He Q. Ren P. Kong X. Xu W. Tang H. Yin Y. (2011). Intrauterine growth restriction alters the metabonome of the serum and jejunum in piglets. *Mol. Biosyst.* 7 2147–2155. 10.1039/c1mb05024a 21584308

[B32] Hill C. J. Lynch D. B. Murphy K. Ulaszewska M. Jeffery I. B. O’Shea C. A. (2017). Erratum to: evolution of gut microbiota composition from birth to 24 weeks in the INFANTMET Cohort. *Microbiome* 5:21 10.1186/s40168-017-0240-3 PMC5240274 28095889

[B33] Hong X. Chen J. Liu L. Wu H. Tan H. Xie G. (2016). Metagenomic sequencing reveals the relationship between microbiota composition and quality of Chinese Rice Wine. *Sci. Rep.* 6:26621 10.1038/srep26621 PMC4886530 27241862

[B34] Hu J. Nie Y. Chen J. Zhang Y. Wang Z. Fan Q. (2016). Gradual changes of gut microbiota in weaned miniature piglets. *Front. Microbiol.* 7:1727 10.3389/fmicb.2016.01727 PMC5090779 27853453

[B35] Hu L. Liu Y. Yan C. Peng X. Xu Q. Xuan Y. (2015). Postnatal nutritional restriction affects growth and immune function of piglets with intra-uterine growth restriction. *Br. J. Nutr.* 114 53–62. 10.1017/S0007114515001579 26059215

[B36] Inman C. F. Haverson K. Konstantinov S. R. Jones P. H. Harris C. Smidt H. (2010). Rearing environment affects development of the immune system in neonates. *Clin. Exp. Immunol.* 160 431–439. 10.1111/j.1365-2249.2010.04090.x 20184618 PMC2883114

[B37] Ivarsson E. Roos S. Liu H. Y. Lindberg J. E. (2014). Fermentable non-starch polysaccharides increases the abundance of *Bacteroides-Prevotella-Porphyromonas* in ileal microbial community of growing pigs. *Animal* 8 1777–1787. 10.1017/S1751731114001827 25046106

[B38] Ivorra C. Garcia-Vicent C. Chaves F. J. Monleon D. Morales J. M. Lurbe E. (2012). Metabolomic profiling in blood from umbilical cords of low birth weight newborns. *J. Transl. Med.* 10:142 10.1186/1479-5876-10-142 PMC3551816 22776444

[B39] Kabat A. M. Srinivasan N. Maloy K. J. (2014). Modulation of immune development and function by intestinal microbiota. *Trends Immunol.* 35 507–517. 10.1016/j.it.2014.07.010 25172617 PMC6485503

[B40] Kang D. W. Park J. G. Ilhan Z. E. Wallstrom G. Labaer J. Adams J. B. (2013). Reduced incidence of *Prevotella* and other fermenters in intestinal microflora of autistic children. *PLoS One* 8:e68322 10.1371/journal.pone.0068322 PMC3700858 23844187

[B41] Kostic A. D. Gevers D. Siljander H. Vatanen T. Hyotylainen T. Hamalainen A. M. (2015). The dynamics of the human infant gut microbiome in development and in progression toward type 1 diabetes. *Cell Host Microbe* 17 260–273. 10.1016/j.chom.2015.01.001 25662751 PMC4689191

[B42] Leduc L. Delvin E. Ouellet A. Garofalo C. Grenier E. Morin L. (2011). Oxidized low-density lipoproteins in cord blood from neonates with intra-uterine growth restriction. *Eur. J. Obstet. Gynecol. Reprod. Biol.* 156 46–49. 10.1016/j.ejogrb.2011.01.007 21324580

[B43] Lei J. Feng D. Zhang Y. Zhao F. Q. Wu Z. San Gabriel A. (2012). Nutritional and regulatory role of branched-chain amino acids in lactation. *Front. Biosci.* 17 2725–2739. 10.2741/4082 22652809

[B44] Li M. Bauer L. L. Chen X. Wang M. Kuhlenschmidt T. B. Kuhlenschmidt M. S. (2012). Microbial composition and in vitro fermentation patterns of human milk oligosaccharides and prebiotics differ between formula-fed and sow-reared piglets. *J. Nutr.* 142 681–689. 10.3945/jn.111.154427 22399522 PMC3301989

[B45] Li N. Wang W. Wu G. Wang J. (2017). Nutritional support for low birth weight infants: insights from animal studies. *Br. J. Nutr.* 117 1390–1402. 10.1017/S000711451700126X 28606217

[B46] Lin G. Liu C. Feng C. Fan Z. Dai Z. Lai C. (2012). Metabolomic analysis reveals differences in umbilical vein plasma metabolites between normal and growth-restricted fetal pigs during late gestation. *J. Nutr.* 142 990–998. 10.3945/jn.111.153411 22513987

[B47] Liu H. Wang J. He T. Becker S. Zhang G. Li D. (2018). Butyrate: a double-edged sword for health? *Adv. Nutr.* 9 21–29. 10.1093/advances/nmx009 29438462 PMC6333934

[B48] Mach N. Berri M. Estelle J. Levenez F. Lemonnier G. Denis C. (2015). Early-life establishment of the swine gut microbiome and impact on host phenotypes. *Environ. Microbiol. Rep.* 7 554–569. 10.1111/1758-2229.12285 25727666

[B49] Maier T. V. Lucio M. Lee L. H. VerBerkmoes N. C. Brislawn C. J. Bernhardt J. (2017). Impact of dietary resistant starch on the human gut microbiome, metaproteome, and metabolome. *mBio* 8:e01343–17 10.1128/mBio.01343-17 PMC5646248 29042495

[B50] Masella A. P. Bartram A. K. Truszkowski J. M. Brown D. G. Neufeld J. D. (2012). PANDAseq: paired-end assembler for illumina sequences. *BMC Bioinformatics* 13:31 10.1186/1471-2105-13-31 PMC3471323 22333067

[B51] Matamoros S. Gras-Leguen C. Le Vacon F. Potel G. de La Cochetiere M. F. (2013). Development of intestinal microbiota in infants and its impact on health. *Trends Microbiol.* 21 167–173. 10.1016/j.tim.2012.12.001 23332725

[B52] Miyazaki M. Kim Y. C. Ntambi J. M. (2001). A lipogenic diet in mice with a disruption of the stearoyl-CoA desaturase 1 gene reveals a stringent requirement of endogenous monounsaturated fatty acids for triglyceride synthesis. *J. Lipid Res.* 42 1018–1024. 11441127

[B53] Mu C. Yang Y. Su Y. Zoetendal E. G. Zhu W. (2017). Differences in microbiota membership along the gastrointestinal tract of piglets and their differential alterations following an early-life antibiotic intervention. *Front. Microbiol.* 8:797 10.3389/fmicb.2017.00797 PMC5422473 28536561

[B54] Naito E. Yoshida Y. Makino K. Kounoshi Y. Kunihiro S. Takahashi R. (2011). Beneficial effect of oral administration of *Lactobacillus casei* strain Shirota on insulin resistance in diet-induced obesity mice. *J. Appl. Microbiol.* 110 650–657. 10.1111/j.1365-2672.2010.04922.x 21281408

[B55] Pop M. Walker A. W. Paulson J. Lindsay B. Antonio M. Hossain M. A. (2014). Diarrhea in young children from low-income countries leads to large-scale alterations in intestinal microbiota composition. *Genome Biol.* 15:R76 10.1186/gb-2014-15-6-r76 PMC4072981 24995464

[B56] Pruesse E. Quast C. Knittel K. Fuchs B. M. Ludwig W. Peplies J. (2007). SILVA: a comprehensive online resource for quality checked and aligned ribosomal RNA sequence data compatible with ARB. *Nucleic Acids Res.* 35 7188–7196. 10.1093/nar/gkm864 17947321 PMC2175337

[B57] Quesnel H. Brossard L. Valancogne A. Quiniou N. (2008). Influence of some sow characteristics on within-litter variation of piglet birth weight. *Animal* 2 1842–1849. 10.1017/S175173110800308X 22444091

[B58] Quesnel H. Farmer C. Devillers N. (2012). Colostrum intake: influence on piglet performance and factors of variation. *Livest. Sci.* 146 105–114. 10.1016/j.livsci.2012.03.010

[B59] Rezaei R. Wang W. Wu Z. Dai Z. Wang J. Wu G. (2013). Biochemical and physiological bases for utilization of dietary amino acids by young Pigs. *J. Anim. Sci. Biotechnol.* 4:7 10.1186/2049-1891-4-7 PMC3599606 23445937

[B60] Ridlon J. M. Kang D. J. Hylemon P. B. (2006). Bile salt biotransformations by human intestinal bacteria. *J. Lipid Res.* 47 241–259. 10.1194/jlr.R500013-JLR200 16299351

[B61] Rooks M. G. Garrett W. S. (2016). Gut microbiota, metabolites and host immunity. *Nat. Rev. Immunol.* 16 341–352. 10.1038/nri.2016.42 27231050 PMC5541232

[B62] Saavedra J. M. Dattilo A. M. (2012). Early development of intestinal microbiota: implications for future health. *Gastroenterol. Clin. North. Am.* 41 717–731. 10.1016/j.gtc.2012.08.001 23101683

[B63] Schloss P. D. Westcott S. L. Ryabin T. Hall J. R. Hartmann M. Hollister E. B. (2009). Introducing mothur: open-source, platform-independent, community-supported software for describing and comparing microbial communities. *Appl. Environ. Microbiol.* 75 7537–7541. 10.1128/AEM.01541-09 19801464 PMC2786419

[B64] Schokker D. Zhang J. Zhang L. L. Vastenhouw S. A. Heilig H. G. Smidt H. (2014). Early-life environmental variation affects intestinal microbiota and immune development in new-born piglets. *PLoS One* 9:e100040 10.1371/journal.pone.0100040 PMC4062469 24941112

[B65] Shi X. Wei X. Yin X. Wang Y. Zhang M. Zhao C. (2015). Hepatic and fecal metabolomic analysis of the effects of *Lactobacillus rhamnosus* GG on alcoholic fatty liver disease in mice. *J. Proteome Res.* 14 1174–1182. 10.1021/pr501121c 25592873 PMC6469358

[B66] Sun Y. Su Y. Zhu W. (2016). Microbiome-metabolome responses in the cecum and colon of pig to a high resistant starch diet. *Front. Microbiol.* 7:779 10.3389/fmicb.2016.00779 PMC4880592 27303373

[B67] Sung M. M. Kim T. T. Denou E. Soltys C. L. M. Hamza S. M. Byrne N. J. (2017). Improved glucose homeostasis in obese mice treated with resveratrol is associated with alterations in the gut microbiome. *Diabetes Metab. Res. Rev.* 66 418–425. 10.2337/db16-0680 27903747

[B68] Turroni S. Brigidi P. Cavalli A. Candela M. (2018). Microbiota-host transgenomic metabolism, bioactive molecules from the inside. *J. Med. Chem.* 61 47–61. 10.1021/acs.jmedchem.7b00244 28745893

[B69] Vaughn P. Thomas P. Clark R. Neu J. (2003). Enteral glutamine supplementation and morbidity in low birth weight infants. *J. Pediatr.* 142 662–668. 10.1067/mpd.2003.208 12838195

[B70] Wang J. Chen L. Li D. Yin Y. Wang X. Li P. (2008). Intrauterine growth restriction affects the proteomes of the small intestine, liver, and skeletal muscle in newborn pigs. *J. Nutr.* 138 60–66. 10.1093/jn/138.1.60 18156405

[B71] Wang J. Feng C. Liu T. Shi M. Wu G. Bazer F. W. (2017). Physiological alterations associated with intrauterine growth restriction in fetal pigs: causes and insights for nutritional optimization. *Mol. Reprod. Dev.* 84 897–904. 10.1002/mrd.22842 28661576

[B72] Wang J. Tang H. Wang X. Zhang X. Zhang C. Zhang M. (2016). The structural alteration of gut microbiota in low-birth-weight mice undergoing accelerated postnatal growth. *Sci. Rep.* 6:27780 10.1038/srep27780 PMC4899793 27277748

[B73] Wang L. Lee Y. K. Bundman D. Han Y. Thevananther S. Kim C. S. (2002). Redundant pathways for negative feedback regulation of bile acid production. *Dev. Cell.* 2 721–731. 10.1016/S1534-5807(02)00187-9 12062085

[B74] Wang M. Radlowski E. C. Monaco M. H. Fahey G. C. Jr. Gaskins H. R. Donovan S. M. (2013). Mode of delivery and early nutrition modulate microbial colonization and fermentation products in neonatal piglets. *J. Nutr.* 143 795–803. 10.3945/jn.112.173096 23616518

[B75] Wang Q. Dong J. Zhu Y. (2012). Probiotic supplement reduces risk of necrotizing enterocolitis and mortality in preterm very low-birth-weight infants: an updated meta-analysis of 20 randomized, controlled trials. *J. Pediatr. Surg.* 47 241–248. 10.1016/j.jpedsurg.2011.09.064 22244424

[B76] Wang X. Lin G. Liu C. Feng C. Zhou H. Wang T. (2014). Temporal proteomic analysis reveals defects in small-intestinal development of porcine fetuses with intrauterine growth restriction. *J. Nutr. Biochem.* 25 785–795. 10.1016/j.jnutbio.2014.03.008 24794015

[B77] Wang X. Wu W. Lin G. Li D. Wu G. Wang J. (2010). Temporal proteomic analysis reveals continuous impairment of intestinal development in neonatal piglets with intrauterine growth restriction. *J. Proteome Res.* 9 924–935. 10.1021/pr900747d 19938879

[B78] Wu G. (2010). Functional amino acids in growth, reproduction, and health. *Adv. Nutr.* 1 31–37. 10.3945/an.110.1008 22043449 PMC3042786

[B79] Wu G. Bazer F. W. Davis T. A. Jaeger L. A. Johnson G. A. Kim S. W. (2007). Important roles for the arginine family of amino acids in swine nutrition and production. *Livest. Sci.* 112 8–22. 10.1016/j.livsci.2007.07.003

[B80] Wu G. Bazer F. W. Johnson G. A. Knabe D. A. Burghardt R. C. Spencer T. E. (2011). Triennial growth symposium: important roles for l-glutamine in swine nutrition and production. *J. Anim. Sci.* 89 2017–2030. 10.2527/jas.2010-3614 21169511

[B81] Wu G. Bazer F. W. Wallace J. M. Spencer T. E. (2006). Board-invited review: intrauterine growth retardation: implications for the animal sciences. *J. Anim. Sci.* 84 2316–2337. 10.2527/jas.2006-156 16908634

[B82] Wu G. Y. (2009). Amino acids: metabolism, functions, and nutrition. *Amino Acids* 37 1–17. 10.1007/s00726-009-0269-0 19301095

[B83] Yang Q. Huang X. Zhao S. Sun W. Yan Z. Wang P. (2017). Structure and function of the fecal microbiota in diarrheic neonatal piglets. *Front. Microbiol.* 8:502 10.3389/fmicb.2017.00502 PMC5364137 28392784

[B84] Yuan T. L. Zhu Y. H. Shi M. Li T. T. Li N. Wu G. Y. (2015). Within-litter variation in birth weight: impact of nutritional status in the sow. *J. Zhejiang Univ. Sci. B* 16 417–435. 10.1631/jzus.B1500010 26055904 PMC4471594

[B85] Zhao W. Wang Y. Liu S. Huang J. Zhai Z. He C. (2015). The dynamic distribution of porcine microbiota across different ages and gastrointestinal tract segments. *PLoS One* 10:e0117441 10.1371/journal.pone.0117441 PMC4331431 25688558

[B86] Zheng J. Xiao X. Zhang Q. Mao L. Yu M. Xu J. (2015). The placental microbiome varies in association with low birth weight in full-term neonates. *Nutrients* 7 6924–6937. 10.3390/nu7085315 26287241 PMC4555154

[B87] Zmora N. Bashiardes S. Levy M. Elinav E. (2017). The role of the immune system in metabolic health and disease. *Cell Metab.* 25 506–521. 10.1016/j.cmet.2017.02.006 28273474

