# Supplementary material for: Differences in the Gut Microbiota Establishment and Metabolome Characteristics Between Low- and Normal-Birth-Weight Piglets During Early-Life
Source: Front Microbiol. 2018 Sep 7;9:1798. doi: 10.3389/fmicb.2018.01798 (PMC6137259; doi:10.3389/fmicb.2018.01798)
Supplement: Supplementary file 1 [file Table_1.doc]

Supplementary Material

# Differences in the gut microbiota establishment and metabolome characteristics between low- and normal-birth-weight piglets during early life

**Na Li, Shimeng Huang, Lili Jiang, Wei Wang, Tiantian Li, Bin Zuo, Zhen Li, Junjun Wang**

*** Correspondence:** Junjun Wang: jkywjj@hotmail.com

# Supplementary Figures and Tables

1. **Supplementary Figures**

**
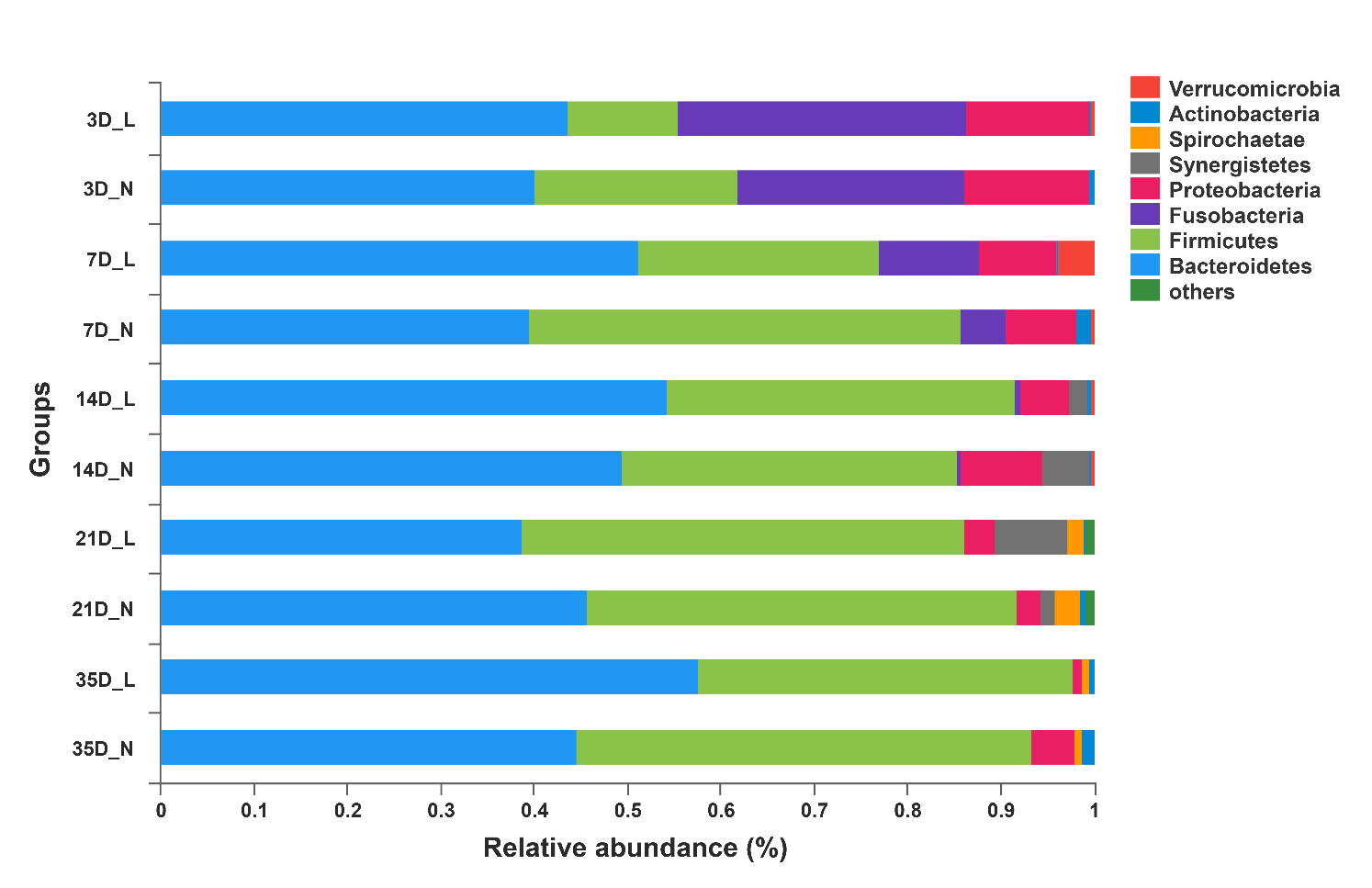
**

**Supplementary Figure S1. Abundant phyla in the gut microbiota of LBW and NBW piglets.** Data are shown as means at each group, *n* = 6 per group. L, low birth weight; N, normal birth weight.

**
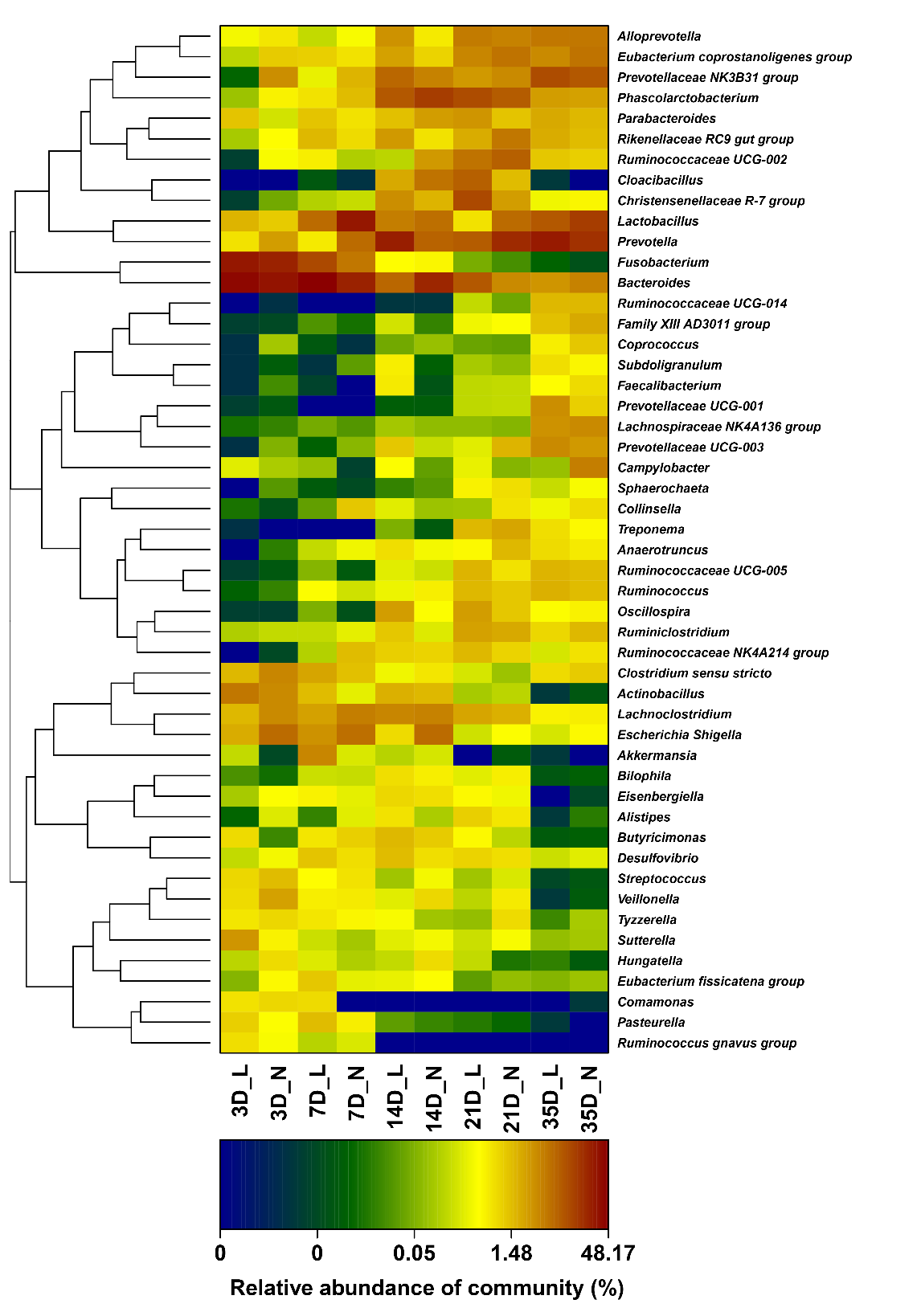
**

**Supplementary Figure S2. The Heatmap of the gut bacterial community structure in LBW and NBW piglets.** The relative abundances of the top 50 taxa at the genus level. Different colors represented the corresponding abundance of each genus, *n* = 6 per group. L, low birth weight; N, normal birth weight.

**
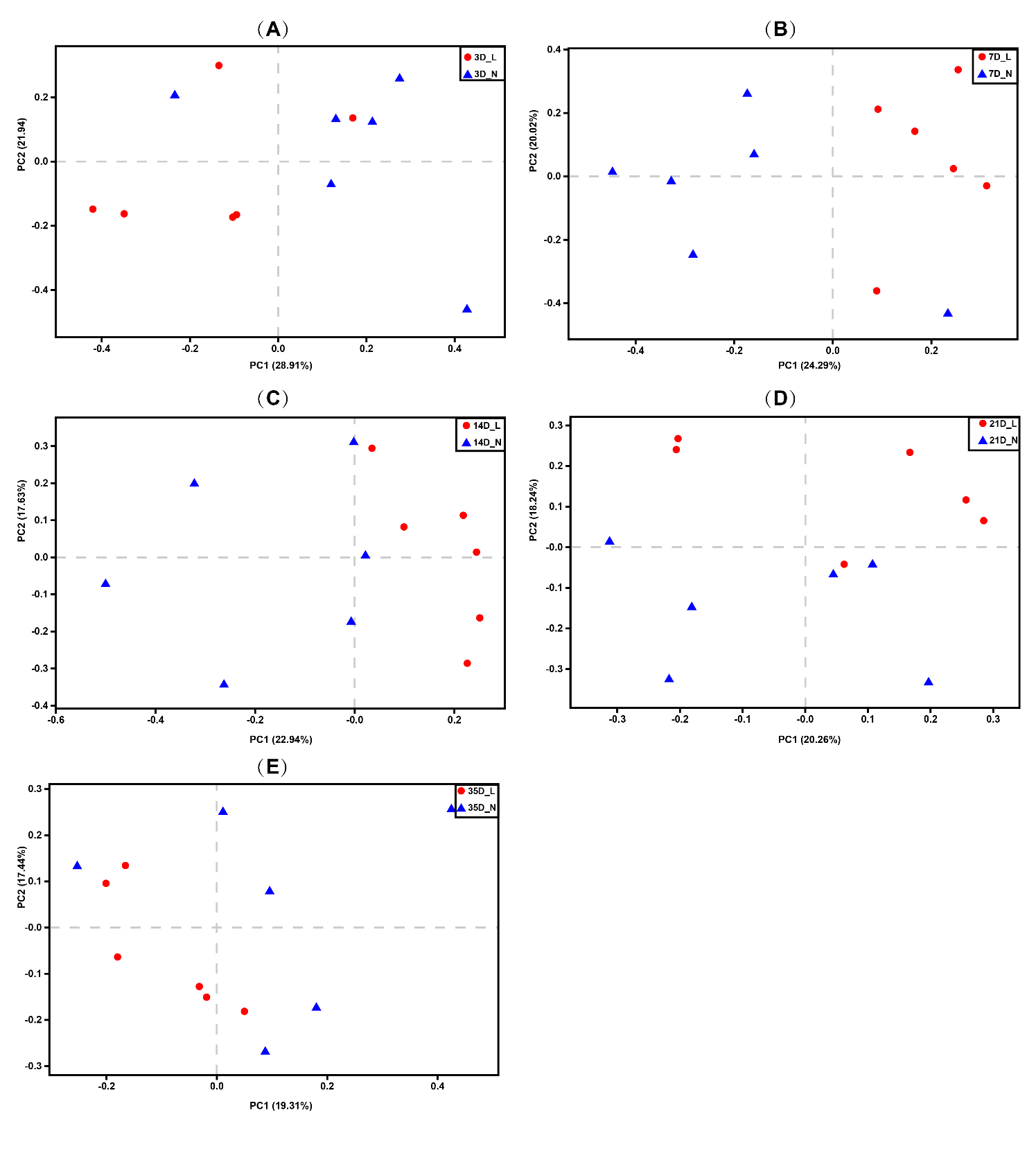
Supplementary Figure S3.** **Principal coordinates analysis (PCoA, Bray-Curtis distance) plot of the gut microbiota community structure between LBW and NBW piglets on D3 (A), D7 (B), D14 (C), D21 (D), and D35 (E).** L, low birth weight; N, normal birth weight. *n* = 6 per group.

**
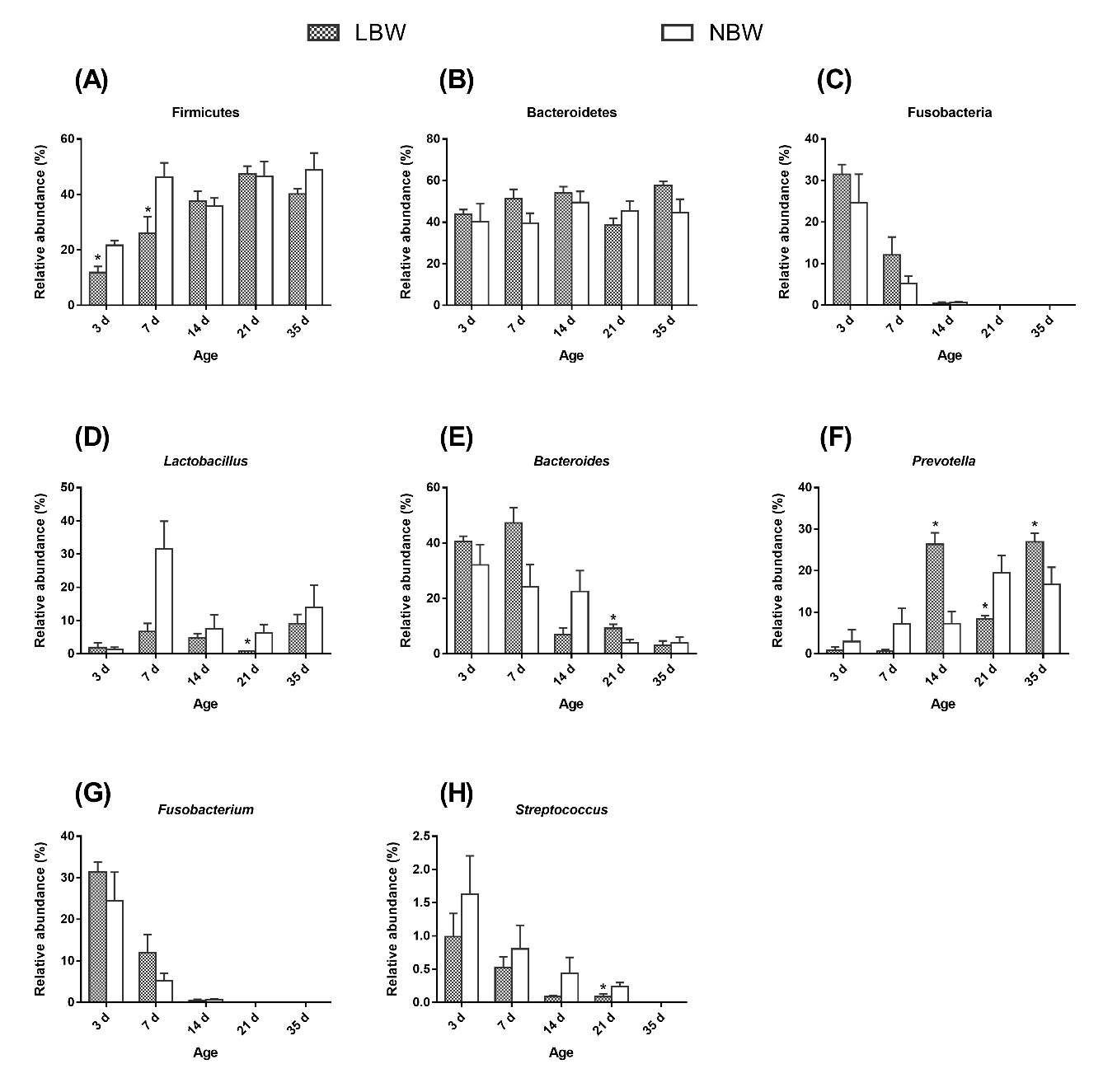
**

**Supplementary Figure S4.** **The differences of 3 key phyla and 5 key genera in the gut bacterial community structure between LBW and NBW piglets.** (A) Firmicutes (B) Bacteroidetes (C) Fusobacteria (D) *Lactobacillus* (E) *Bacteroides* (F) *Prevotella* (G) *Fusobacterium* (H) *Streptococcus.* Data are shown as mean±SEM. Asterisks indicate significant different in LBW piglets from NBW piglets (Mann-Whitney *U*-test and LEfSe analysis), *n* = 6 per group. LBW, low birth weight; NBW, normal birth weight.

# Supplementary Tables

**Supplementary Table** **S1** Growth performance of LBW and NBW piglets^a^

| Item | LBW | | | | |  | NBW | | | | | SEM | *P* value | | |
| --- | --- | --- | --- | --- | --- | --- | --- | --- | --- | --- | --- | --- | --- | --- | --- |
|  | D3 | D7 | D14 | D21 | D35 |  | D3 | D7 | D14 | D21 | D35 |  | Age | BiW | Age*BiW |
| BW(kg) | 1.14 | 1.80 | 2.93 | 4.61 | 6.69 |  | 1.82 | 2.79 | 4.79 | 6.92 | 8.06 | 0.30 | <0.0001 | <0.0001 | 0.006 |
| ADG (g/d) | 86.08 | 132.47 | 147.57 | 179.44 | 165.48 |  | 137.17 | 195.83 | 241.44 | 262.82 | 190.69 | 8.10 | <0.0001 | <0.0001 | 0.292 |

^a^ The results were analyzed using the General Lineal Model (GLM) procedures and presented as Means. *n* = 6 per group. LBW, low birth weight; NBW, normal birth weight; BW, body weight; ADG, average daily gain; BiW, birth weight.

**Supplementary Table** **S2 Genus-level differences between LBW and NBW piglets at each time-point**

| Phylum | Genus | D3 | |  | D7 | |  | D14 | |  | D21 | |  | D35 | |
| --- | --- | --- | --- | --- | --- | --- | --- | --- | --- | --- | --- | --- | --- | --- | --- |
|  |  | LBW | NBW |  | LBW | NBW |  | LBW | NBW |  | LBW | NBW |  | LBW | NBW |
| Bacteroidetes | *Bacteroides* | 40.509 | 32.082 |  | 47.299 | 24.216 |  | 6.995 | 22.532 |  | 9.160* | 3.923 |  | 3.098 | 3.972 |
| Firmicutes | *Lactobacillus* | 1.882 | 1.260 |  | 6.704 | 31.499 |  | 4.709 | 7.550 |  | 0.790* | 6.316 |  | 9.119 | 14.035 |
| Bacteroidetes | *Prevotella* | 0.829 | 2.938 |  | 0.642 | 7.205 |  | 26.343* | 7.276 |  | 8.342* | 19.499 |  | 26.948* | 16.736 |
| Proteobacteria | *Escherichia-Shigella* | 2.061 | 6.809 |  | 3.447 | 6.074 |  | 0.956* | 6.577 |  | 0.196 | 0.478 |  | 0.241 | 0.542 |
| Bacteroidetes | *Alloprevotella* | 0.406 | 0.816 |  | 0.166 | 0.459 |  | 3.288* | 0.709 |  | 4.771 | 4.033 |  | 5.165 | 5.242 |
| Firmicutes | *Christensenellaceae R-7 group* | 0.001 | 0.041 |  | 0.129 | 0.175 |  | 3.521 | 1.873 |  | 12.065* | 2.851 |  | 0.373 | 0.530 |
| Bacteroidetes | *Prevotellaceae UCG-003* | 0.001 | 0.058 |  | 0.006 | 0.062 |  | 1.289* | 0.157 |  | 0.287* | 1.786 |  | 3.547 | 2.663 |
| Firmicutes | *Oscillospira* | 0.001 | 0.001 |  | 0.048 | 0.003 |  | 2.623* | 0.463 |  | 2.499 | 1.175 |  | 0.434 | 0.566 |
| Firmicutes | *Ruminiclostridium* | 0.118 | 0.179 |  | 0.187 | 0.333 |  | 1.275* | 0.286 |  | 2.499 | 2.170 |  | 0.944 | 1.660 |
| Bacteroidetes | *Butyricimonas* | 0.921 | 0.016 |  | 0.810 | 1.091 |  | 1.581 | 1.146 |  | 0.481* | 0.148 |  | 0.004 | 0.004 |
| Firmicutes | *Ruminococcaceae UCG-005* | 0.001 | 0.003 |  | 0.059* | 0.004 |  | 0.289 | 0.186 |  | 1.754* | 0.749 |  | 1.748 | 1.500 |
| Proteobacteria | *Campylobacter* | 0.286 | 0.115 |  | 0.074* | 0.001 |  | 0.442 | 0.028 |  | 0.326* | 0.048 |  | 0.074 | 4.744 |
| Firmicutes | *Streptococcus* | 0.990 | 1.625 |  | 0.524 | 0.805 |  | 0.087 | 0.440 |  | 0.087* | 0.243 |  | 0.001 | 0.003 |
| Firmicutes | *Hungatella* | 0.144 | 0.923 |  | 0.290 | 0.114 |  | 0.171 | 0.818 |  | 0.139 | 0.009 |  | 0.012* | 0.004 |
| Firmicutes | *Faecalibacterium* | 0.001* | 0.018 |  | 0.001 | 0.000 |  | 0.710* | 0.003 |  | 0.148 | 0.179 |  | 0.499 | 0.830 |
| Firmicutes | *Hydrogenoanaerobacterium* | 0.004 | 0.021 |  | 0.178 | 0.104 |  | 0.704 | 0.233 |  | 0.225 | 0.365 |  | 0.000* | 0.018 |
| Firmicutes | *Ruminococcaceae UCG-004* | 0.008 | 0.019 |  | 0.148 | 0.139 |  | 0.142 | 0.202 |  | 0.365 | 0.388 |  | 0.098* | 0.410 |
| Firmicutes | *Intestinimonas* | 0.004 | 0.003 |  | 0.026 | 0.031 |  | 0.781* | 0.096 |  | 0.411 | 0.358 |  | 0.001 | 0.001 |
| Firmicutes | *Flavonifractor* | 0.134 | 0.368 |  | 0.063* | 0.314 |  | 0.382 | 0.057 |  | 0.008 | 0.286 |  | 0.018 | 0.025 |
| Firmicutes | *Butyricicoccus* | 0.209 | 0.017 |  | 0.513 | 0.035 |  | 0.053 | 0.031 |  | 0.077* | 0.031 |  | 0.085* | 0.489 |
| Bacteroidetes | *Odoribacter* | 0.000 | 0.032 |  | 0.000 | 0.013 |  | 0.264 | 0.518 |  | 0.000* | 0.102 |  | 0.001 | 0.001 |
| Firmicutes | *Oscillibacter* | 0.001 | 0.000 |  | 0.003 | 0.000 |  | 0.029* | 0.003 |  | 0.200 | 0.034 |  | 0.375 | 0.205 |
| Firmicutes | *Candidatus Soleaferrea* | 0.000 | 0.000 |  | 0.001 | 0.000 |  | 0.001 | 0.000 |  | 0.083* | 0.001 |  | 0.230 | 0.253 |
| Firmicutes | *Howardella* | 0.016 | 0.002 |  | 0.095 | 0.020 |  | 0.020 | 0.052 |  | 0.008* | 0.051 |  | 0.012* | 0.055 |
| Proteobacteria | *Helicobacter* | 0.000 | 0.018 |  | 0.039 | 0.002 |  | 0.016 | 0.001 |  | 0.031* | 0.005 |  | 0.043 | 0.106 |
| Firmicutes | *Anaerofilum* | 0.002 | 0.004 |  | 0.004 | 0.001 |  | 0.008* | 0.000 |  | 0.017 | 0.007 |  | 0.033 | 0.066 |
| Firmicutes | *Moryella* | 0.000 | 0.000 |  | 0.000 | 0.000 |  | 0.020* | 0.000 |  | 0.059* | 0.014 |  | 0.017 | 0.041 |
| Proteobacteria | *Oxalobacter* | 0.000 | 0.005 |  | 0.001 | 0.001 |  | 0.017 | 0.012 |  | 0.028* | 0.009 |  | 0.032 | 0.028 |

The relative abundances (%) are shown as means, *n* = 6 per group. Asterisks indicate significant different in LBW piglets from NBW piglets (LEfSe analysis). LBW, low birth weight; NBW, normal birth weight.

**Supplementary Table S3 Differentially abundant metabolites in feces between LBW and NBW piglets at each time-point**

| Age (d) | Metabolites | m/z | Formula | *P* value | Fold change^1^ | The changes in LBW piglets compared with NBW ones | Related pathway |
| --- | --- | --- | --- | --- | --- | --- | --- |
| *3* | Glycocholic acid | 466.3154 | C_26_H_43_NO_6_ | 0.002 | 2.58 | Up-regulated | Primary bile acid biosynthesis |
| *3* | L-Valine | 118.0862 | C_20_H_41_NO_3_ | 0.050 | 2.16 | Up-regulated | Valine, leucine and isoleucine biosynthesis |
| *3* | Vanilpyruvic acid | 193.0493 | C_10_H_10_O_5_ | 0.047 | 2.19 | Up-regulated | Aromatic L-amino acid decarboxylase deficiency |
| *7* | Linoleic acid | 298.2735 | C_18_H_32_O_2_ | 0.049 | 2.43 | Up-regulated | Linoleic acid metabolism |
| *7* | α-Linolenic acid | 261.2207 | C_18_H_30_O_2_ | 0.041 | 2.61 | Up-regulated | α-Linolenic acid metabolism |
| *7* | Palmitic acid | 239.2364 | C_16_H_32_O_2_ | 0.007 | 2.26 | Up-regulated | Fatty acid metabolism |
| *7* | Indoleacetic acid | 176.0703 | C_10_H_9_NO_2_ | 0.022 | 7.61 | Up-regulated | Tryptophan metabolism |
| *7* | α-dimorphecolic acid | 279.2311 | C_18_H_32_O_3_ | 0.014 | 6.10 | Up-regulated | Linoleic acid metabolism |
| *7* | Cyclohexaneundecanoic acid | 269.2468 | C_17_H_32_O_2_ | 0.006 | 2.04 | Up-regulated | A minor constituent of human milk free fatty acids and triglyceride fractions |
| *7* | 5,8-Tetradecadienoic acid | 207.1739 | C_14_H_24_O_2_ | 0.004 | 1.95 | Up-regulated | Unsaturated fatty acid oxidation |
| *7* | 3-Oxododecanoic acid | 197.1531 | C_12_H_22_O_3_ | 0.040 | 2.03 | Up-regulated | Fatty acid biosynthesis |
| *7* | Cyclohexanecarboxylic acid | 129.0907 | C_7_H_12_O_2_ | 0.007 | 1.79 | Up-regulated | Microbial metabolism |
| *7* | N^1^-Acetylspermine | 245.2331 | C_12_H_28_N_4_O | 0.005 | 0.10 | Down-regulated | The conversion of spermine to spermidine |
| *7* | N-Undecanoylglycine | 226.1795 | C_13_H_25_NO_3_ | 0.014 | 0.45 | Down-regulated | Fatty acid oxidation |
| *7* | N-Acetylcadaverine | 145.1333 | C_7_H_16_N_2_O | 0.008 | 0.09 | Down-regulated | The breakdown of amino acids |
| *14* | Oleic acid | 283.2624 | C_18_H_34_O_2_ | 0.047 | 0.42 | Down-regulated | Fatty acid biosynthesis |
| *14* | Linoleic acid | 263.2362 | C_18_H_32_O_2_ | 0.046 | 0.40 | Down-regulated | Linoleic acid metabolism |
| *14* | Kynurenic acid | 190.0497 | C_10_H_7_NO_3_ | 0.001 | 0.10 | Down-regulated | Tryptophan metabolism |
| *14* | Indoleacetic acid | 176.0703 | C_10_H_9_NO_2_ | 0.042 | 0.45 | Down-regulated | Tryptophan metabolism |
| *14* | 2-Phenylacetamide | 118.0650 | C_8_H_9_NO | 0.049 | 0.56 | Down-regulated | Phenylalanine metabolism |
| *14* | Deoxycholic acid | 375.2884 | C_24_H_40_O_4_ | 0.022 | 0.51 | Down-regulated | Secondary bile acid biosynthesis |
| *14* | Tetracosahexaenoic acid | 339.2675 | C_24_H_36_O_2_ | 0.010 | 0.37 | Down-regulated | α-Linolenic Acid and Linoleic Acid Metabolism |
| *14* | Myristoleic acid | 227.1999 | C_14_H_26_O_2_ | 0.048 | 0.42 | Down-regulated | Fatty acid metabolism |
| *14* | 3-Oxotetradecanoic acid | 225.1843 | C_14_H_26_O_3_ | 0.033 | 0.20 | Down-regulated | Fatty acid biosynthesis |
| *14* | Cyclohexanecarboxylic acid | 129.0907 | C_7_H_12_O_2_ | 0.042 | 0.42 | Down-regulated | Microbial metabolism |
| *21* | 25-Hydroxycholesterol | 403.3565 | C_27_H_46_O_2_ | 0.007 | 7.63 | Up-regulated | Primary bile acid biosynthesis |
| *21* | Chenodeoxycholic acid | 375.2883 | C_24_H_40_O_4_ | 0.012 | 2.17 | Up-regulated | Primary bile acid biosynthesis |
| *21* | Arachidonic acid | 287.2363 | C_20_H_32_O_2_ | 0.014 | 2.00 | Up-regulated | Arachidonic acid metabolism |
| *21* | Stearoylcarnitine | 428.3727 | C_25_H_49_NO_4_ | 0.032 | 1.49 | Up-regulated | Mitochondrial Beta-Oxidation of Long Chain Saturated Fatty Acids |
| *21* | Zymosterol intermediate 2 | 367.3355 | C_27_H_44_O | 0.029 | 4.65 | Up-regulated | Steroid biosynthesis |
| *21* | Docosahexaenoic acid | 329.2465 | C_22_H_32_O_2_ | 0.015 | 1.68 | Up-regulated | Biosynthesis of unsaturated fatty acids |
| *21* | Desaminotyrosine | 184.0968 | C_9_H_10_O_3_ | 0.046 | 1.54 | Up-regulated | Metabolism of flavonoids and amino acids by enteric bacteria |
| *21* | Desmosterol | 385.3457 | C_27_H_44_O | 0.037 | 0.44 | Down-regulated | Steroid biosynthesis |
| *21* | Phenylalanylphenylalanine | 295.1436 | C_18_H_20_N_2_O_3_ | 0.032 | 0.31 | Down-regulated | The precursor for the amino acid tyrosine |
| *21* | 3-Oxohexadecanoic acid | 288.2528 | C_16_H_30_O_3_ | 0.017 | 0.34 | Down-regulated | Fatty acid biosynthesis |
| *35* | Etiocholanedione | 306.2421 | C_19_H_28_O_2_ | 0.041 | 2.18 | Up-regulated | Steroid hormone biosynthesis |
| *35* | 3-Hydroxyhippuric acid | 178.0496 | C_9_H_9_NO_4_ | 0.035 | 1.95 | Up-regulated | Fatty acid metabolism |
| *35* | L-Phenylalanine | 148.0754 | C_9_H_11_NO_2_ | 0.046 | 2.09 | Up-regulated | Phenylalanine, tyrosine and tryptophan biosynthesis |
| *35* | Palmitic acid | 239.2363 | C_16_H_32_O_2_ | 0.028 | 0.56 | Down-regulated | Fatty acid metabolism |
| *35* | L-Glutamic acid | 148.0602 | C_5_H_9_NO_4_ | 0.013 | 0.77 | Down-regulated | Arginine and proline metabolism |
| *35* | Succinic acid | 101.0236 | C_4_H_6_O_4_ | 0.0050 | 0.58 | Down-regulated | Alanine, aspartate and glutamate metabolism |
| *35* | 3β,7α-Dihydroxy-5-cholestenoate | 415.3197 | C_27_H_44_O_4_ | 0.013 | 0.78 | Down-regulated | Primary bile acid biosynthesis |
| *35* | Allolithocholic acid | 359.2938 | C_24_H_40_O_3_ | 0.027 | 0.42 | Down-regulated | A bile acid present in normal serum and feces |
| *35* | N-Acetylneuraminic acid | 348.0692 | C_11_H_19_NO_9_ | 0.035 | 0.22 | Down-regulated | Amino Sugar Metabolism |
| *35* | Hypogeic acid | 272.2579 | C_16_H_30_O_2_ | 0.006 | 0.71 | Down-regulated | Fatty acid metabolism |
| *35* | N-Acetylserine | 130.0499 | C_5_H_9_NO_4_ | 0.013 | 0.79 | Down-regulated | Acetylation of the N-terminal amino acid |

^1^Fold change was calculated by dividing the mean of normalized intensity of each plasma metabolite in the LBW piglets by the mean of normalized intensity of each plasma in the NBW ones. Fold change > 1 indicates that the value for LBW piglets is greater than the NBW ones, whereas the opposite is indicated by a fold change < 1. *n* = 6 per group. LBW, low birth weight; NBW, normal birth weight.

**
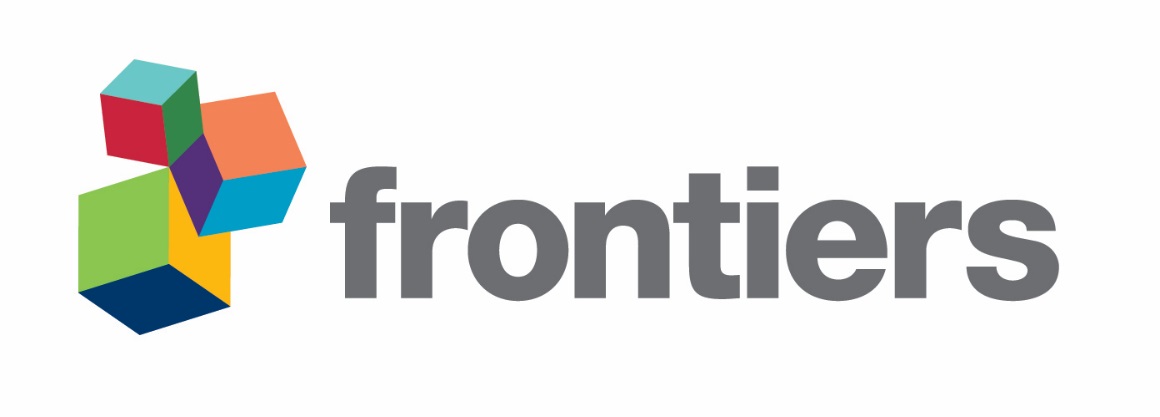
**
